# Plasma Virome of HIV-infected Subjects on Suppressive Antiretroviral Therapy Reveals Association of Differentially Abundant Viruses with Distinct T-cell Phenotypes and Inflammation

**DOI:** 10.2174/0113892029279786240111052824

**Published:** 2024-01-22

**Authors:** Tannu Bhagchandani, Mohammad M. Ul Haque, Shilpa Sharma, Md Zubbair Malik, Ashwini K. Ray, Urvinder S. Kaur, Ankita Rai, Anjali Verma, Kamal K. Sawlani, Rupesh Chaturvedi, Himanshu Dandu, Abhishek Kumar, Ravi Tandon

**Affiliations:** 1 Laboratory of AIDS Research and Immunology, School of Biotechnology, Jawaharlal Nehru University, New Delhi, India;; 2 School of Computational and Integrative Sciences, Jawaharlal Nehru University, New Delhi, India;; 3 School of Biotechnology, Jawaharlal Nehru University, New Delhi, India;; 4 Host-Pathogen Interaction Laboratory, School of Biotechnology, Jawaharlal Nehru University, New Delhi, India;; 5 Laboratory of Metabolic Disorder and Environmental Biotechnology, Department of Environmental Studies, Faculty of Science, University of Delhi, New Delhi, India;; 6 Department of Medicine, King George’s Medical University, Lucknow, India;; 7 Special Centre for System Medicine, Jawaharlal Nehru University, New Delhi, India;; 8 Institute of Bioinformatics, International Technology Park, Bangalore; India;; 9 Manipal Academy of Higher Education (MAHE), Manipal, India

**Keywords:** Plasma virome, HIV, ART, *Human gammaherpesvirus 4*, T cell exhaustion, inflammation

## Abstract

**Background:**

The plasma virome represents the overall composition of viral sequences present in it. Alteration in plasma virome has been reported in treatment naïve and immunocompromised (CD4 count < 200) people with HIV (PWH). However, the effect of ART on virome composition in PWH on ART with preserved CD4 counts is poorly understood.

**Objectives:**

We aimed to assess the alterations in plasma virome in PWH on ART in comparison to HIV-negative uninfected controls and to further investigate possible associations of plasma viruses with inflammation and immune dysfunction, namely, immunosenescence and immune exhaustion.

**Methods:**

Plasma viral DNA from PWH on ART and controls was used for sequencing on the Illumina Nextseq500 platform, followed by the identification of viral sequences using an automated pipeline, VIROMATCH. Multiplex cytokine assay was performed to measure the concentrations of various cytokines in plasma. Immunophenotyping was performed on PBMCs to identify T cell markers of immunosenescence and immune exhaustion.

**Results:**

In our observational, cross-sectional pilot study, chronically infected PWH on ART had significantly different viral species compositions compared to controls. The plasma virome of PWH showed a significantly high relative abundance of species *Human gammaherpesvirus 4,* also known as Epstein-Barr virus (EBV). Moreover, EBV emerged as a significant viral taxon differentially enriched in PWH on ART, which further correlated positively with the exhaustion phenotype of T cells and significantly increased TNF-α in PWH on ART. Additionally, a significantly increased proportion of senescent T cells and IL-8 cytokine was detected in PWH on ART.

**Conclusion:**

Altered plasma virome influenced the inflammatory response and T-cell phenotype in PWH on ART.

## 1 INTRODUCTION

The virome is part of the metagenome that consists of the genome or gene fragments of viruses. These can be both DNA and RNA because viruses have both DNA and RNA genomes [1, 2]. Approximately 300 billion viruses are present on or inside an adult human body. The human virobiota is made up of eukaryotic and prokaryotic viruses, which differ based on their host and the virus class they include [1-4]. Eukaryotic viruses are those viruses that replicate in the cells of eukaryotic hosts, and they include DNA, RNA, retroviruses and newly discovered giant viruses, whereas prokaryotic viruses are those that replicate in the cells of prokaryotic hosts, and they include DNA and RNA viruses [5]. The eukaryotic viruses present in the human body can be pathogenic or non-pathogenic. Pathogenic viruses can cause acute or chronic infections [2, 6]. Viruses that cause acute infections in the host have a short incubation period during which they produce many viral copies and are also cleared in a short time by the host immune system [7]. Whereas viruses that cause chronic infections reside in the host for a long time. They can persist either by continuous replication or by latency. Viruses like HIV, HBV, and HCV persist in the host by continually producing their viral copies, which keep triggering infections that last long. While some viruses like HIV, EBV, *etc*., remain transcriptionally silent until reactivated by some stimulus and hence are present in the absence of clinical manifestations [8]. However, some eukaryotic viruses are non-pathogenic and they spend longer durations in the body without causing infections [9]. Likewise, a large fraction of prokaryotic viruses, mainly bacteriophages that make up the majority of human virome are also regarded as non-pathogenic as they replicate in bacterial cells [10]. These non-pathogenic eukaryotic viruses and bacteriophages present in a healthy, asymptomatic individual in the absence of observable infections or symptoms make up the healthy human virome and are known as resident or commensal viruses [1, 4, 10, 11]. The composition and abundance of these viruses vary from site to site, and they also change during immunosuppression and diseases such as Crohn’s disease, ulcerative colitis, Type 1 Diabetes Mellitus (DM), T2DM, obesity, asthma, chronic obstructive pulmonary disease (COPD), hypertension, HIV *etc* [2, 6, 11-18].

HIV infects and progressively depletes CD4-positive T cells. If not treated, it further progresses to AIDS after passing acute and chronic stages of infection. Although ART is being used, HIV remains a global concern [19, 20]. The leaky gut during HIV infection leads to microbial translocation, which brings about TLR-mediated systemic inflammation and immune activation [21, 22]. Alterations in the number and composition of gut microbial communities during HIV infection have been reported by several groups in the past [23-25]. In addition to this, altered virome composition and abundance also contribute to HIV-AIDS-associated enteropathy and inflammation [26]. The commensal virome composition and diversity changes during HIV infection owing to the deterioration of the immune system due to a distinctive reduction in the number of CD4 T cells [27]. A plasma virome-based study reported a significant increase in reads belonging to Anelloviruses and HERVs in AIDS patients with CD4 count < 20 cells/µL than HIV-infected patients with CD4 count > 700 cells/µL [28]. Furthermore, another study showed an increased percentage of bacteriophages and HERVs in the plasma of HIV-positive patients with CD4 count < 125 cells/µL as opposed to healthy controls [29].

All these studies suggest shifts in enteric and plasma virome in treatment naïve HIV-infected individuals or in patients with a compromised immune system. Less is known about the plasma virome composition in HIV-infected individuals undergoing ART and with preserved CD4 counts. Owing to the leaky gut phenomenon in HIV, we hypothesise that along with bacteria, some viruses may also escape from the lumen of the gut into the body’s systemic circulation, where they may either cause infection by challenging the immune system or may be present commensally in the blood or plasma by stimulating low-level immune responses without causing any observable symptoms.

During HIV infection, the immune system is constantly exposed to HIV antigens that gradually lead to inflammation and immune dysfunction, namely, immunosenescence and immune exhaustion. Immunosenescence is defined as an age-related decline in the function of both innate and adaptive components of the immune system [30]. Immune exhaustion is defined as the reduction in function of T-cells in a stepwise manner, ultimately leading to their death. It is characterized by the constitutive expression of inhibitory receptors like PD-1, TIM-3 and CTLA-4 on T cells [31]. Low-level virome in the plasma may add to the persistent immune activation and inflammation in the absence of active HIV replication, which may, in turn, result in premature immune senescence and immune exhaustion over time, despite viral suppression by ART.

We have analyzed plasma virome composition and its relative abundance using high throughput sequencing in PWH on ART and compared them with that of uninfected controls. In addition, we have investigated the possible association of virome composition with inflammation and immune dysfunction, namely, immunosenescence and immune exhaustion. To the best of our knowledge, this is the first virome study being conducted in ART-suppressed Indian subjects living with HIV.

## 2 MATERIALS AND METHODS

### 2.1 Study Participants

This is an observational, cross-sectional pilot study that included 9 chronically infected PWH on ART and 8 age- matched uninfected controls above the age of eighteen. To study plasma virome, 6 samples from each group were randomly chosen. The study population included those PWH who were on ART for more than 1.5 years and had preserved CD4 counts and undetectable viral load (VL < 50 copies/mL) in their plasma. PWH included here were either on TLE (tenofovir + lamivudine + efavirenz) or ZLN (zidovudine + lamivudine + nevirapine) drugs at the time of sample collection. Patients with co-infections like TB, HCV and HBV were excluded from the study. The controls were age and sex-matched to PWH. All those individuals without HIV, HBV, HCV and TB infections were included as controls. The information for CD4 count and viral load were obtained from the ART center, King George’s Medical University (KGMU), Lucknow. Tests for plasma viral load and CD4 counts were performed at KGMU in accordance with the guidelines of the National AIDS Control Organisation (NACO). The plasma viral load was measured using Abbott RealTime HIV-1 Assay on m20000 instrument (Abbott), with a lower limit of detection of 50 copies of RNA/mL. CD4 counts were estimated using the CD4% easy kit with subsequent enumeration on the Cyflow Counter system (Sysmex Partec GmbH). The details of the participants are listed in Tables **1** and **S1**. Samples were obtained after receiving written informed consent from participants and Institutional Ethics Review Board (IERB) approval from Jawaharlal Nehru University (JNU), New Delhi and King George’s Medical University (KGMU), Lucknow. The study was conducted under the approved guidelines by the Institutional Biosafety Committee (IBSC) of JNU.

### 2.2 Sample Collection and Processing

Approximately 5-6 ml of the peripheral blood withdrawn from recruited participants was collected in EDTA-coated tubes and centrifuged at 2,000 rpm for 15 minutes to separate the plasma, which was stored at -80°C till further use. As per the manufacturer's protocol, PBMCs were isolated by density gradient centrifugation using the HiSep LSM 1077 (Himedia, India) and were cryopreserved in liquid nitrogen after resuspending the cells in freezing media (10% DMSO in FBS).

### 2.3 Viral DNA Extraction

The participants were chosen randomly for plasma virome sequencing. Plasma samples stored at −80°C were thawed on ice, and around 200 µL was used for nucleic acid extraction. According to the manufacturer's guidelines, viral DNA was extracted from plasma samples using the commercially available QIAampMinElute Virus Spin Kit (Qiagen). This kit isolates both DNA and RNA simultaneously. The quantity and purity of isolated nucleic acids were assessed by measuring absorbance at 260 and 280 nm on a nanodrop spectrophotometer.

### 2.4 Virome Sequencing

Viral nucleic acid was sequenced in 3 steps: quality check, library preparation and sequencing. Viral nucleic acid was quantified using Nanodrop and Qubit Fluorometer. Samples with A_260/280_ < 2.2 were passed for further processing. After quality check, the Illumina TruSeq Nano DNA Library Prep Kit was used to create paired-end (PE) sequencing libraries. Quantity and quality of PCR-enriched PE libraries were checked on the 4200 Tape Station system (Agilent Technologies) using high sensitivity D1000 screen tape as per manufacturer instructions. After obtaining the Qubit concentration of the libraries and mean peak sizes from the Tape station profile, PE Illumina libraries were loaded on the Illumina Nextseq500 platform for cluster generation and PE sequencing using 2x150 base pair chemistry. In PE sequencing, the template fragments are sequenced in both the forward and reverse directions.

### 2.5 Bioinformatic Analysis

The raw PE reads were screened for the presence of viral reads using a published pipeline, VIROMATCH, against the RefSeq viral database collected from NCBI Gen Bank. Reads were processed by VIROMATCH in three key steps- 1. Read preparation and host read filtration. 2. Mapping of reads to a viral reference genome. 3. Validation mapping to NCBI references. The first step included the pairing of reads, removal of adapters, trimming of low-quality base pairs (phred score < 33), filtration of reads post-trimming (minimum length applied = 50), removal of short reads (readlen < 50), masking of low complexity and repetitive base pairs and removal of reads mapping to the host genome. In the second step, non-host reads were mapped to virus-only nucleotide and translated nucleotide databases. Blast hits with an e-value less than 0.01 were collected, and unmapped reads were removed (e-value = 0.01). In the last step, candidate viral reads obtained were validated by mapping to NCBI nucleotide (nt) and nonredundant (nr) amino acid databases. Sequences with unambiguous mapping to viral databases were considered viral hits. The best hits were collected (pid < 0.15 and pidprox < 0.04). Finally, it provided reports detailing viral taxonomic classification and quantification of mapped reads [32]. The read counts obtained in the final table were considered the absolute abundance of the viruses in downstream analysis.

### 2.6 Immunophenotyping

Cryopreserved PBMC samples were thawed at 37°C in a water bath and washed with Flow Cytometry Staining buffer (FCSB) (PBS, 0.02% EDTA and 1% BSA). Approximately 0.5x10^6^ cells per well were seeded in a 96-well V-bottom plate. Further, cells were surface stained for several markers by incubating them with antibodies for half an hour on ice. This was followed by washing cells twice with FCSB and then finally fixing them with 2% Paraformaldehyde (PFA) on ice. Live/Dead aqua-amine-reactive dye (AARD, Invitrogen) was used for Live/Dead staining. Fluorescently labeled monoclonal antibodies were used for staining cell surface markers in two different panels. Panel 1 included senescence markers with PerCP-Cy5.5-anti-CD3, FITC-anti-CD4, APC-anti-CD8, PE/Cy7-anti-CD28, and PE-anti-CD57 antibodies. Panel 2 included exhaustion markers that had PerCP-Cy5.5-anti-CD3, APC-anti-CD4, APC-Cy7-anti-CD8, BV421-anti-PD-1, BB515-anti-TIM-3 and PE-anti-CTLA-4 antibodies. Fluorescence minus one (FMO) controls were prepared for gating CD28, CD57, PD-1, TIM-3 and CTLA-4 positive populations. To avoid fluorescence spillover, compensation controls were prepared using a BD Comp bead set according to manufacturer instructions (BD Anti-Mouse Ig, k/Negative Compensation Particles set, Becton, Dickinson and company).

### 2.7 Flow Cytometry Analysis

The data was acquired on BD FACS Aria Fusion^TM^ and analyzed using FACS Diva and FlowJo software. The following gating strategy was used to identify desired cells. Singlets were gated using an FSC-A and FSC-H plot. Lymphocytes were identified using FSC-A and SSC-A. This was followed by gating the live cells (AARD -ve cells). Further, CD4 and CD8-positive T-cells were identified after gating live CD3 cells. Finally, senescent and exhausted cells were gated based on FMO controls and the frequency of cells was estimated using FlowJO v.10. The gating strategy is shown in Figs. (**S1** and **S2**).

### 2.8 Multiplex Cytokine Assay

The concentration of cytokines present in the plasma was examined by a Luminex 17-Plex assay (Bio-Rad). The plasma samples were thawed on ice and centrifuged at 1000 rpm for 15 minutes. The supernatant was used to prepare a 1:4 dilution of plasma for a volume of 120 µL. Then, 50 µL of diluted plasma samples were added to the wells in duplicate. The remaining steps were followed as written in the manufacturer’s protocol. The assay measured plasma levels of the following 17 cytokine/chemokine: Hu G-CSF, Hu GM-CSF, Hu IFN-g, Hu IL-1b, Hu IL-2, Hu IL-4, Hu IL-5, Hu IL-6, Hu IL-7, Hu IL-8, Hu IL-10, Hu IL-12 (p70), Hu IL-13, Hu IL-17, Hu MCP-1 (MCAF), Hu MIP-1b, Hu TNF-alpha. Cytokine standards and each sample were tested in duplicate. Data was acquired on the Bio-Plex 200 system using Bio-Plex manager software, v4.1 (Bio-Rad).

### 2.9 Diversity Analysis

The absolute read counts of viral species obtained after taxonomy assignment and abundance estimation were loaded into R, and reads were normalized by Total Sum Scaling (TSS), also known as relative abundance, using the microbiomeMarker package in R [33]. TSS normalizes count data by dividing feature read counts by the total number of reads in each sample. The read count of each virus in each sample was divided by the total number of viral read counts in that sample. The stacked bar plots of relative abundance were generated using the Phyloseq package in R [34]. Alpha diversity, richness, evenness and beta diversity were estimated using the Microbiome package in R [35]. Here, alpha diversity was calculated using the Shannon-Weaver index, richness was estimated using the Chao1 index and evenness was assessed using the Simpson index [36]. Further, Bray-Curtis dissimilarity was utilized to compute beta diversity, which was then employed in Principal Coordinate Analysis (PCoA) to create ordination plots [36]. Ordination plots were made using the ggPlot2 package in R [37].

### 2.10 Statistical Analysis

The statistical difference in virome composition between PWH and controls was calculated by applying the Adonis test using the Vegan package in R [38]. The difference in the relative abundance of viruses was estimated using the Mann-Whitney test in GraphPad Prism v.6 (Graph/Pad Prism version 6.0.0 for Windows, GraphPad Software, San Diego, California USA, www.graphpad.com). Differential abundance analysis was performed using the Analysis of Compositions of Microbiomes with Bias Correction (ANCOM-BC) package in R [39]. Spearman rank correlation test was performed using the package Hmisc in R [40]. Correlation graphs were plotted using the package corrplot in R [41]. Further, the difference in immune cell populations and plasma cytokine concentrations between PWH and controls were estimated using the Mann-Whitney test in GraphPad Prism v.6 (GraphPad Prism version 6.0.0 for Windows, GraphPad Software, San Diego, California USA, www. graphpad.com).

## 3 RESULTS

### 3.1 Composition of Plasma Virome in PWH on ART and Uninfected Controls

**Fig. (1):**
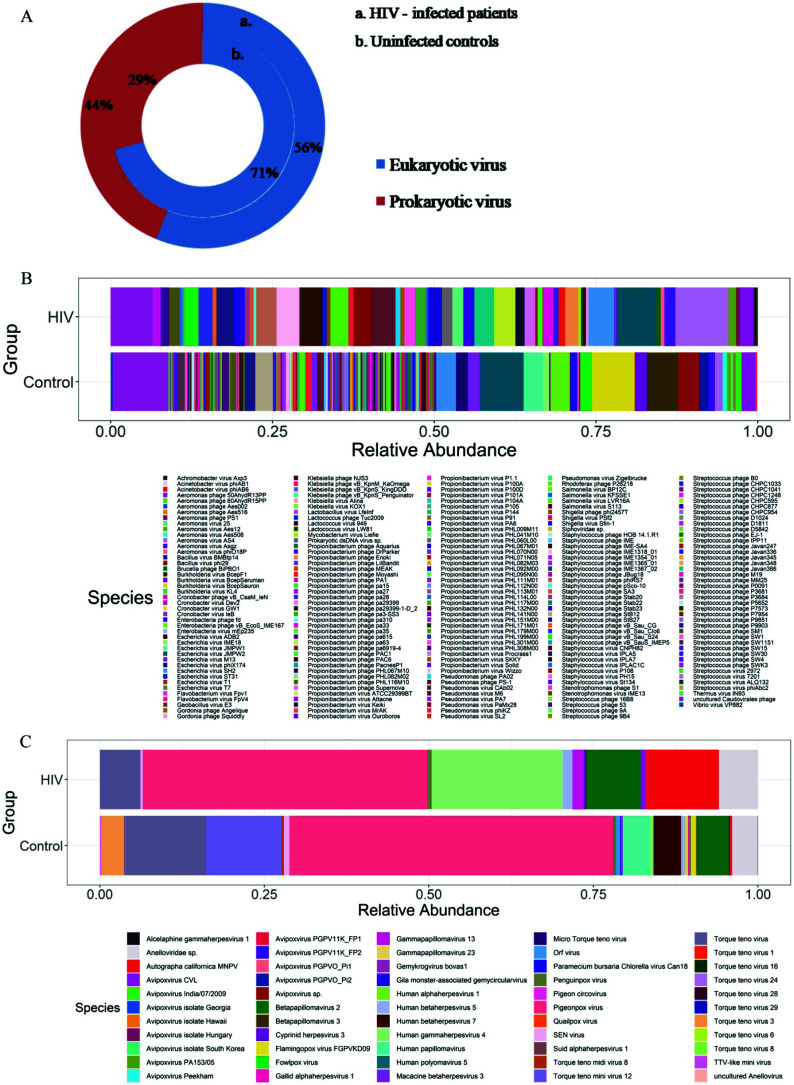
(**A**) Doughnut representing the overall percentage of eukaryotic and prokaryotic viruses in PWH on ART and uninfected controls. Stacked bar plots showing the relative abundance of (**B**) prokaryotic and (**C**) eukaryotic viral species present in PWH on ART and uninfected controls. The X-axis represents two groups - control and HIV, and the Y-axis represents relative abundance.

Plasma virome analysis resulted in an average of 5,026,946 reads per sample. These reads were bioinformatically processed through VIROMATCH, which yielded 21,806 non-ambiguous viral reads in total, after removing short reads, reads that map to the human genome and ambiguous reads. Finally, the total number of viral species identified in our study is 268. Out of these, 213 are prokaryotic viruses, and 55 are eukaryotic viruses with different percentages in PWH and uninfected controls (Fig. **1A**). Their relative abundance in PWH and controls is shown by stacked bar plots (Figs. **1B** and **C**). To understand the complete landscape of viruses among all samples, heatmaps showing the relative abundances of all the prokaryotic and eukaryotic viral species identified in individual samples were plotted (Fig. **S3**). Except for one viral species- Prokaryotic ds DNA virus sp, all other prokaryotic viruses identified in our study are bacteriophages. More than 97% of the bacteriophages identified belong to order *Caudovirales*. Despite the large number of bacteriophages identified, only a few phages, namely, *Aeromonas virus AS4*, *Staphylococcus* *phage phi RS7*, *Escherichia virus JMPW2*, *Escherichia virus SH2*, *Pseudomonas* *phage PS-1*, *Staphylococcus* *phage Stb27* and *Staphylococcus* *phage IME1318_01* are shared among all or some samples. A large number of phages are specific to one or two individuals. The human eukaryotic viruses identified are Anelloviruses, Torque teno viruses, *Human Papillomavirus* and Polyomavirus. Various non-human eukaryotic viruses infecting plants, animals and insects were also identified in our study. Overall, the highly abundant viral reads found in HIV-infected subjects and control belong to families *Anelloviridae, Herpesviridae, unclassified Caudovirales, Poxviridae, Myoviridae, Drexlerviridae, Papillomaviridae, Baculoviridae, Siphoviridae* and *Herelleviridae*. The stacked bar plots and heatmaps of viral families are shown as Figs. (**S4A**, **B**, **C** and **D**).

### 3.2 PWH on ART Show Distinct Pattern of Plasma Virome Compared to Uninfected Controls

**Fig. (2):**
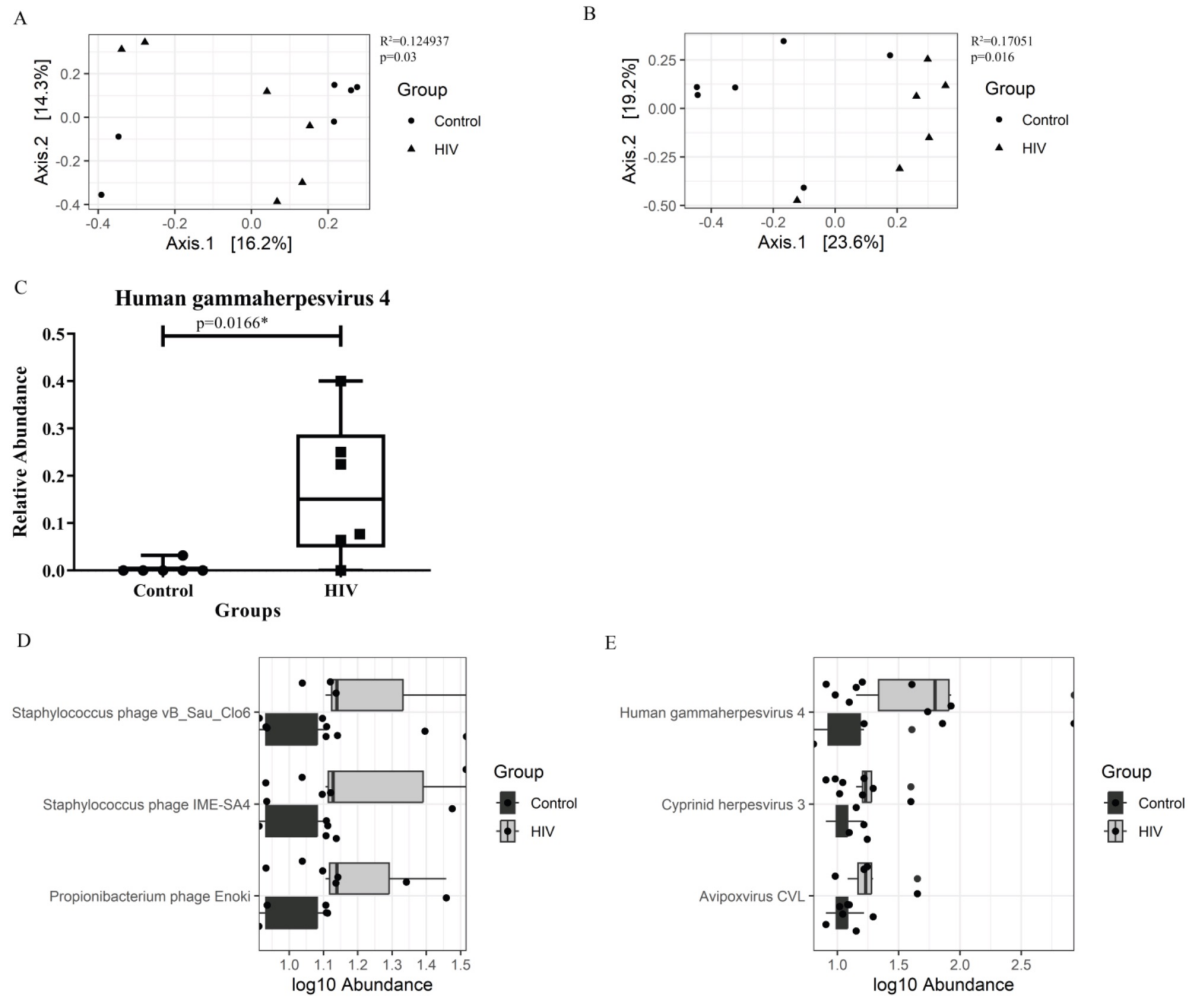
Ordination plots representing beta diversity of (**A**) prokaryotic and (**B**) eukaryotic viral species. Principal Component Analysis (PCoA) based on Bray-Curtis dissimilarities representing viral dissimilarity on each axis. Here, maximum variability is summarized by the axes 1 & 2. Percent in axes is the variation percentage at each axis. Statistical significance was tested using the Adonis test. R^2^ represents virome composition variance and *p* < 0.05 was considered to be significant. (**C**) Box plot representing difference in the relative abundance of viral species *Human gammaherpesvirus 4* between PWH on ART and uninfected controls. The X-axis shows the different groups- control and HIV. The Y-axis shows the relative abundance of the taxa. Mann-Whitney-Wilcoxon test was used to evaluate statistical significance, *p* < 0.05 was considered to be significant. Viral abundance box plots representing differentially enriched (**D**) prokaryotic and (**E**) eukaryotic viral species between PWH on ART and uninfected controls. The X-axis represents log_10_ abundance of viral species and Y-axis represents differentially abundant viral species. Only those differential species with *p* < 0.05 and W statistic > 2 are shown.

In our analysis, no significant difference in alpha diversity, richness and evenness was observed for prokaryotic and eukaryotic viral species. Further, using PCoA, the dissimilarity in the prokaryotic and eukaryotic plasma viral communities between PWH on ART and uninfected controls was assessed. Here, Bray-Curtis dissimilarity was used to determine beta diversity, which was further used in PCoA analysis for generating ordination plots where maximum variability is summarized by axes 1 & 2 in percentage. It was found that the prokaryotic and eukaryotic plasma viral species of HIV-infected subjects on ART differed significantly from those of controls (R^2^ = 0.124937, *p* = 0.03 and R^2^ = 0.17051, *p* = 0.016, respectively, Figs. **2A** and **B**). As a result of HIV infection, a variation of 12.49% and 17.05% in virome composition was observed between PWH on ART and controls for prokaryotic and eukaryotic viral species, respectively. The ordination plots of viral families are shown as Figs. (**S4E** and **F**).

### 3.3 Increased Relative Abundance of *Human Gammaherpesvirus 4* in PWH on ART Compared to Uninfected Controls

To understand the differences in the plasma virome, PWH on ART and uninfected controls were compared for the relative abundance of all prokaryotic and eukaryotic viral species. Our research showed a significant rise in the relative abundance of the viral species *Human gammaherpesvirus 4* in PWH on ART (*p* = 0.0166, Fig. **2C**).

### 3.4 Differentially Abundant Viral Taxa between PWH on ART and Uninfected Controls

To determine the viral taxa linked to HIV, differential abundance analysis was performed using ANCOM-BC for prokaryotic and eukaryotic viruses. The results revealed a significant increase in log_10_ abundance of prokaryotic viral species- *Staphylococcus* *phage vB Clo6*, *Staphylococcus* *phage IME-SA4*, *Propionibacterium* *phage Enoki* in PWH on ART. Among the eukaryotic viral species, log_10_ abundance of *Human gammaherpesvirus 4*, Cyprinid herpesvirus 3 and *Avipoxvirus CVL* was found to be increased significantly in PWH on ART (Figs. **2D** and **E**). Differential taxa with *p* value less than 0.05 and W statistic greater than 2 are listed in Table **S2**.

### 3.5 Increased CD57^+^ CD8 T Cells and Pro-inflammatory Cytokines TNF-α and IL-8 in PWH on ART in Comparison to Uninfected Controls

**Fig. (3):**
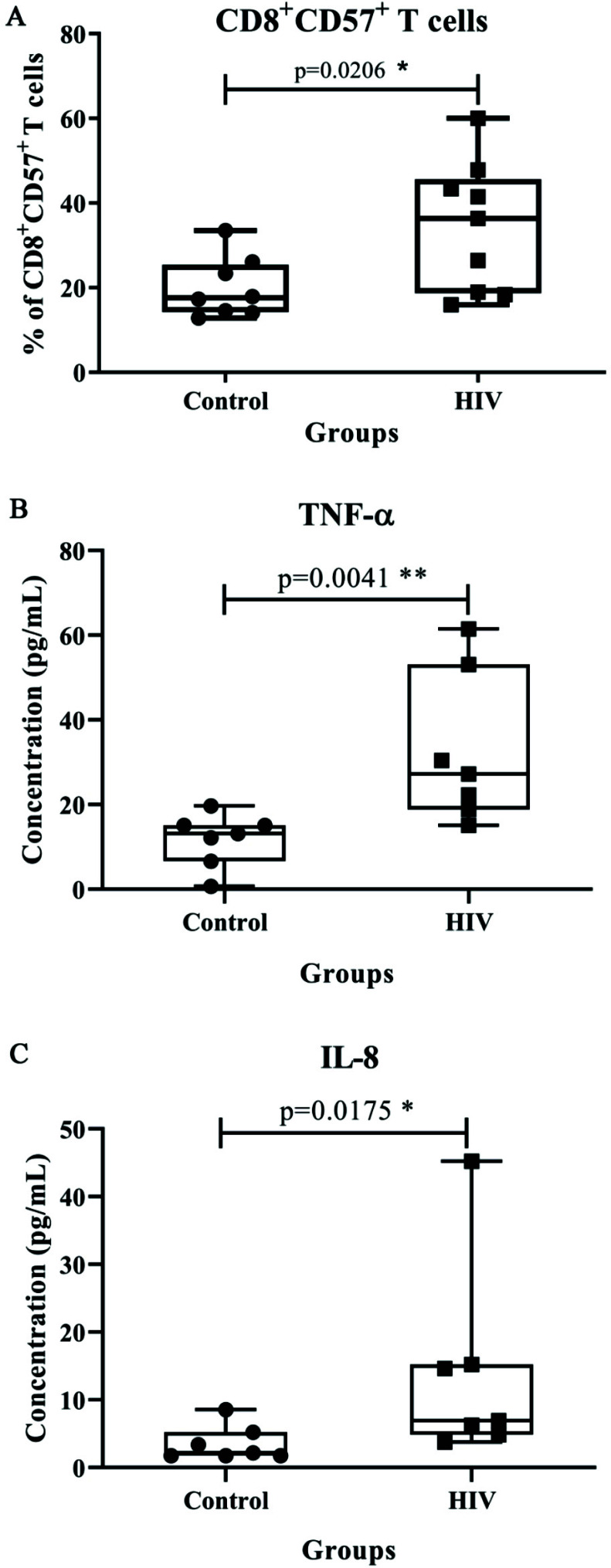
Box plots representing difference in the (**A**) frequency of CD57^+^ CD8^+^ T cells, the plasma concentration of cytokines (**B**) TNF-α and (**C**) IL-8 between PWH on ART and uninfected controls. The X-axis shows the different groups - control and HIV. The Y-axis shows the frequency of CD57^+^ CD8^+^ T cells in percentage and plasma concentration of cytokines in pg/mL. Mann-Whitney-Wilcoxon test was used to evaluate statistical significance, *p* < 0.05 was considered to be significant.

To understand the impact of viral sequences on immune cells, we first characterized T cells for the presence of senescence and exhaustion markers in PWH on ART and compared their results to those of uninfected controls. When compared to controls, PWH on ART displayed significantly higher CD57^+^CD8^+^T cell expression (17.61%; 14.23, 25.44 *versus* 36.35%; 18.68, 45.61) (*p* = 0.0206, Fig. **3A**). No significant differences were found for the exhaustion phenotype of the T cells between PWH on ART and uninfected controls (data not shown). Further, multiplex cytokine assay revealed high expression levels of TNF-α and IL-8 in plasma of PWH on ART as compared to uninfected controls ((13.16 pg/mL; 6.650, 15.11 *versus* 27.24 pg/mL; 53.12, 18.8) and (2.19 pg/mL; 1.76, 5.23 *versus* 6.95 pg/mL; 4.87, 15.24), respectively) (*p* = 0.0041 and *p* = 0.0175, respectively) (Figs. **3B** and **C**). No significant difference was obtained for the plasma concentrations of other cytokines (data not shown). Additionally, immune exhaustion and T cell senescence in the lymphocyte population (CD4 and CD8) are shown Fig. (**S5**). A significant difference was observed in the percentage of the senescent marker (CD57) present on T cell populations (CD4 and CD8 T cells) between uninfected controls and PWH on ART. Therefore, in PWH, T cell senescence was more prominent, as also demonstrated above in the case of CD8 T cells as a separate cell type (Fig. **3A**).

### 3.6 Plasma Virome is Significantly Associated with Immune Dysfunction Phenotype and Inflammation in PWH on ART

**Fig. (4):**
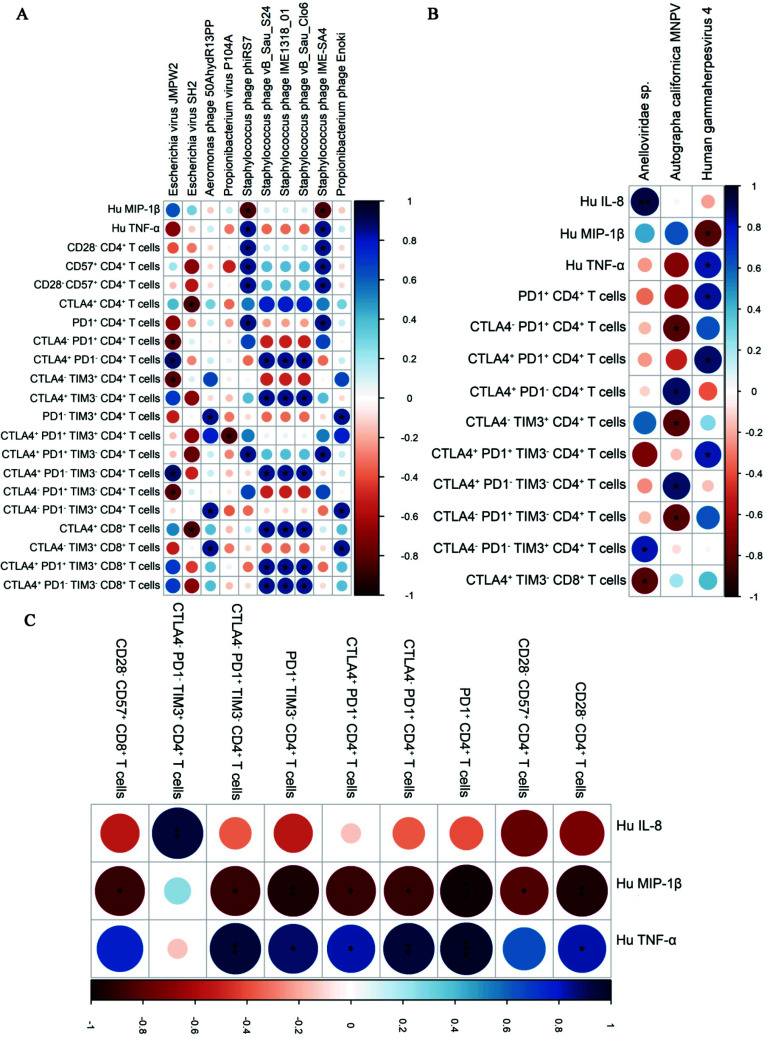
Correlograms representing correlation patterns of (**A**) prokaryotic and (**B**) eukaryotic viral species with T cell immune phenotypes and inflammatory cytokines and of (**C**) immune cell phenotypes with inflammatory cytokines in PWH on ART. Spearman’s Rank Correlation test was used to measure the association between parameters on the X and Y axes. Asterisks here are used to indicate correlations that are significant (*p* < 0.05), *P* < 0.05*, < 0.01**, < 0.001***. Only statistically significant correlations were plotted, where blue circles denote direct correlations and red circles denote inverse correlations. The size and shading show the correlation's strength, with darker shades and larger circles denoting stronger correlations than light small ones.

**Fig. (5):**
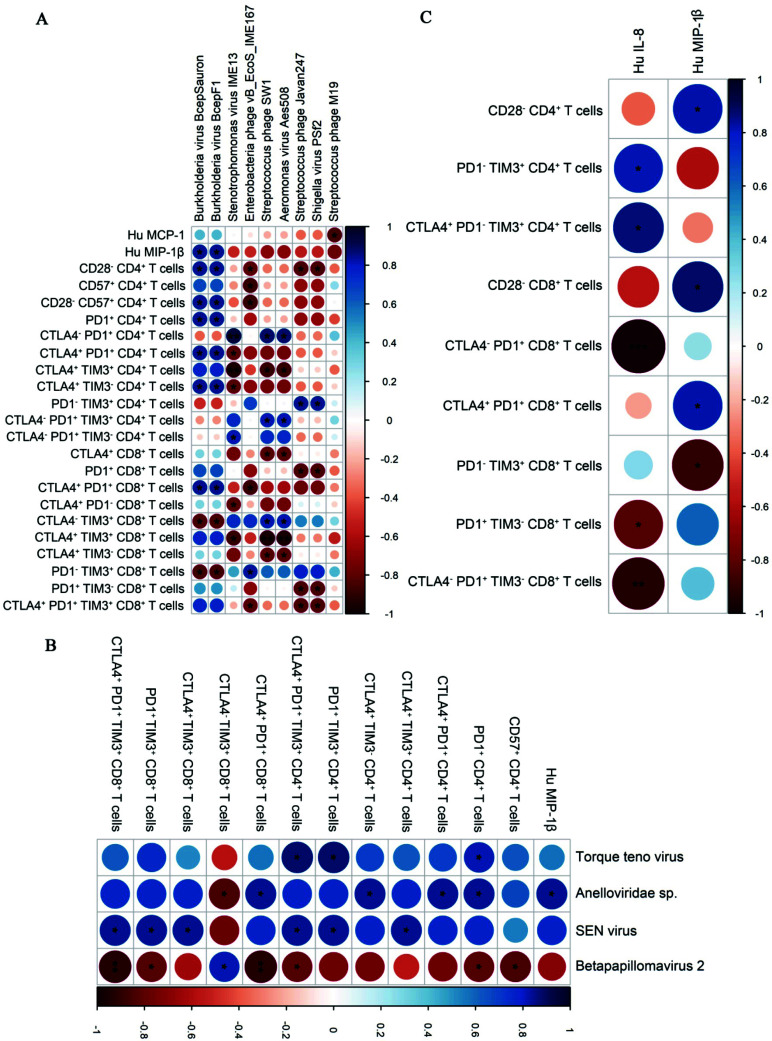
Correlograms representing correlation patterns of (**A**) prokaryotic and (**B**) eukaryotic viral species with T cell immune phenotypes and inflammatory cytokines and of (**C**) immune phenotypes and inflammatory cytokines in uninfected controls. Spearman’s Rank Correlation test was used to measure the association between parameters on the X and Y axes. Asterisks here are used to indicate correlations that are significant (*p* < 0.05), *P* < 0.05*, < 0.01**, < 0.001***. Only statistically significant correlations were plotted, where blue circles denote direct correlations and red circles denote inverse correlations. The size and shading show the correlation's strength, with darker shades and larger circles denoting stronger correlations than light, small ones.

To understand the effect of differentially abundant (DA) viruses on inflammation and immune cells, the relationship of all prokaryotic and eukaryotic viral species with inflammation, immunosenescence and immune exhaustion phenotype was characterized by evaluating their correlation patterns in PWH on ART and uninfected controls. We identified direct correlation patterns of the differentially abundant prokaryotic - *Staphylococcus* *phage vB Clo6*, *Staphylococ cus* *phage IME-SA4* and *Propionibacterium* *phage Enoki*; and eukaryotic viral species- *Human gammaherpesvirus 4*, autographa californica multiple nucleopolyhedrovirus with cytokines and T cell phenotypes of senescence and exhaustion in PWH on ART (Figs. **4A** and **B**). None of these species showed significant correlation patterns in uninfected controls. In particular, *Human gammaherpesvirus 4* correlated positively with exhausted T cells (PD1^+^ CD4^+^, CTLA4^+^PD1^+^ CD4^+^, CTLA4^+^PD1^+^TIM3^-^ CD4^+^T cells) and cytokine Hu TNF-α in PWH on ART (Fig. **4B**). Additionally, TNF-α correlated positively with exhausted T cells in PWH on ART (Fig. **4C**). Several viral species also showed direct and indirect correlation patterns with markers of immune dysfunction in uninfected controls (Figs. **5A** and **B**). Further, cytokines IL-8 and MIP-1b showed direct and indirect correlation patterns with markers of immune dysfunction in uninfected controls (Fig. **5C**). The correlation patterns of viral families with cytokines and immune phenotypes are shown in Fig. (**S6**). A list of significant correlations at species and family level are presented in Tables **S3****-****12** respectively.

## 4 DISCUSSION

The metagenomic study of the plasma virome of PWH on ART and controls revealed the presence of 268 viruses. Those were broadly divided into prokaryotic and eukaryotic viruses for further analysis, as both interact with the immune system differently. Besides the viruses routinely found in humans, some eukaryotic viruses infecting plants, insects and animals were also obtained. The presence of these non-human viruses may reflect environmental contamination during extraction and sequencing [6]. Further, the percentage of prokaryotic viruses, mainly bacteriophages, was found to be increased in PWH. As bacteriophages interact with bacteria and the presence of bacteriophages reflects the presence of their host bacteria, this increase in phage population could be due to the increased intestinal permeability of bacteria and bacterial elements in the systemic circulation of PWH [10, 21, 29].

Despite the increased percentage of prokaryotic viruses observed in PWH, no difference in diversity, richness and evenness of prokaryotic viruses between PWH on ART and controls was observed. This may be because the composition of bacteriophages is not properly shared among the groups, and rather, they show a great extent of inter-individual variability. No difference in diversity, richness and evenness of eukaryotic viruses was observed, possibly because, except a few, most of the viruses were present in very low abundance across all samples. Although a significant difference in eukaryotic and prokaryotic plasma virome compositions based on Bray-Curtis dissimilarity was observed between PWH on ART and uninfected controls, the virome of PWH and controls are poorly grouped into clusters. This suggests that ART can partially restore the plasma virome composition, possibly due to partial restoration of CD4 counts and immune competency in PWH on ART [42, 43].

Alterations in the plasma virome composition during HIV have been documented in AIDS patients, intravenous drug users (IDUs), treatment naïve HIV patients with impaired CD4 counts and in HIV positive MSM (Men who have sex with Men) on ART with CD4 counts > 200 [26, 28, 29, 43, 44]. To the best of our knowledge, there is no study reported for heterosexual HIV patients accessing ART and with preserved CD4 counts. Further, HIV infection causes immune activation and inflammation that sustains even during ART. This results in an impaired immune system that, in turn, opens the window for opportunistic and commensal viruses to flourish in ways different from those of healthy people [10, 30]. Therefore, certain prokaryotic and eukaryotic viral species were found to be enriched and associated with HIV in our study.

PWH on ART had a much higher relative abundance of the viral species *Human gammaherpesvirus 4* than uninfected controls. Also, *Human gammaherpesvirus 4* emerged as the differentially abundant species in PWH on ART through the ANCOM-BC test. The incidence of EBV is higher in HIV-infected patients [45]. The presence of the viral species *Human gammaherpesvirus 4* in PWH on ART is substantiated by past research showing increased levels of EBV DNA copies in PBMCs and plasma of ART-suppressed HIV patients measured using PCR [46-48]. Besides EBV, Cyprinid herpesvirus 3 and *Avipoxvirus CVL* were also found to be differentially abundant in PWH on ART. Their role in human infections is not known yet.

The prokaryotic viruses identified to be differentially abundant in PWH on ART are- *Staphylococcus* *phage vB Clo6*, *Staphylococcus* *phage IME-SA4* and *Propionibacterium* *phage Enoki*. These bacteriophages were also observed to be significantly associated with inflammation and T-cell phenotypes of senescence and exhaustion. *Staphylococcus* *phage vB Clo6*, also known as *Staphylococcus aureus* *phage vB Clo6*, infects *Staphylococcus aureus* bacteria, a common cutaneous bacterial pathogen. The prevalence of this bacteria increases in HIV patients and is a major cause of bacterial infections in them [49, 50]. Therefore, the differential abundance of reads belonging to *Staphylococcus* *phage vB Clo6* in PWH in our results could be attributed to the increased abundance of *Staphylococcus aureus* bacteria. *Staphylococcus* *phage IME-SA4*, also known as *Staphylococcus Haemolyticus* *bacteriophage IME-SA4*, infects *Staphylococcus Haemolyticus*, an important hospital pathogen frequently present in human blood. Its association with HIV is unknown [51, 52]. *Propionibacterium* *phage Enoki*, also known as *Propionibacterium acnes* *bacteriophage Enoki*, infects *Cutibacterium acnes* bacteria. This bacteria has been reported to be responsible for immune recovery folliculitis (IRF), an inflammatory disorder in treatment naïve and ART-experienced HIV patients. In a recent study, *Cutibacterium acnes*-related brain abscess has also been reported in an HIV-infected patient [53, 54]. Hence, the differential abundance of reads belonging to *Propionibacterium* *phage Enoki* in PWH in our results could be attributed to the prevalence of its host during HIV infection. Hence, no direct correlation between phages and immune dysfunction phenotypes has been reported in previous studies. We hypothesize that the presence of these differentially abundant phages in PWH could be due to the increased abundance or prevalence of their bacterial hosts in systemic circulation during HIV infection.

Furthermore, high immune activation and inflammation during HIV infection lead to early immunosenescence. Despite suppression of HIV RNA replication and immune reconstitution in HIV-infected patients on ART, there is evidence of immunosenescence, possibly due to the elevation of systemic inflammatory markers [55-57]. Hence, in our study, significantly elevated senescent phenotype (CD57^+^ CD8^+^ T cells) and inflammatory markers- TNF-α and IL-8 in PWH on ART is in line with previous reports [58-61]. Further, during acute infections, the immune checkpoint inhibitors (ICIs) are expressed transiently on the surface of T cells post their activation. During chronic infections like HIV, the ICIs remain elevated due to constant exposure of T cells to viral antigens [31]. However, the expression of ICIs such as PD-1 and TIM-3 decreased in HIV-infected patients undergoing ART due to reduced plasma viral load owing to suppression of viral replication by ART [62-65]. Likewise, we did not observe any significant differences in the exhaustion phenotype of T cells between PWH on ART and controls.

*Human gammaherpesvirus 4*, also known as Epstein-Barr virus (EBV), is a herpesvirus belonging to the family *Herpesviridae.* EBV infects monocytes, T cells, B cells *etc* [66-68]. The LMP-1 gene of EBV triggers NF-kB signalling mediated TNF-α secretion [69, 70]. In general, EBV causes an asymptomatic primary infection in immunocompetent hosts, which is usually controlled by the immune system [68]. However, higher EBV load leads to lymphomas in immunosuppressed individuals and HIV patients by TNF-mediated polyclonal activation of B cells induced either directly by HIV-1 proteins or indirectly by immune activation through TLR-mediated recognition of translocated microbial products, *etc* [71-74]. ART has no effect on EBV DNA load, and rather, EBV is shown to associate with LPS and pro-inflammatory cytokines (IL-6, IL-10 and TNF-α) in ART-suppressed HIV patients with good immunological status [74-76]. In our study, *Human gammaherpesvirus 4* significantly correlated directly with TNF-α in PWH on ART.

To establish lymphomas, EBV suppresses immune response by up-regulating PD-1/PD-L1 on infected cells [66, 72, 77, 78]. A recent finding showed the correlation of EBV DNA levels at year 1 post ART with markers of exhaustion (CD4^+^PD1^+^) and immune activation [79]. Similarly, in our analysis, *Human gammaherpesvirus 4* significantly correlated directly with the exhaustion phenotypes- PD1^+^ CD4^+^, CTLA4^+^PD1^+^ CD4^+^, CTLA4^+^PD1^+^TIM3^-^ CD4^+^ T cells in PWH on ART. During chronic infections such as HIV, elevated levels of TNF-α may induce the expression of PD-1 on infected cells. Blockage of TNFR signaling has confirmed the association between TNF-α and immune exhaustion [80, 81]. Our analysis also showed significant positive correlations between TNF-α and exhaustion phenotypes- PD1^+^ CD4^+^, PD1^+^TIM3^-^ CD4^+^, PD1^+^CTLA4^-^ CD4^+^, PD1^+^CTLA4^+^ CD4^+^, PD1^+^CTLA4^-^TIM3^-^ CD4^+^ T cells.

The major components of blood and plasma virome are viruses belonging to the Anelloviridae family. An increase in the abundance of Anelloviruses, mainly *Torque teno virus* (TTV), is reported in AIDS patients, IDUs, and patients with CD4 counts less than 200 [44, 82]. In addition, TTV viremia is reported to decrease with the introduction of ART and with the increase in CD4 T-cell counts [83, 84]. Likewise, we did not observe any significant difference in the abundance of TTVs in PWH in our study because the PWH included in our study have been on suppressive therapy for more than 1.5 years and have preserved CD4 counts > 200. Further, a plasma virome-based study on MSM has shown a significant increase in the relative abundance of Anellovirus in ART-treated HIV-positive MSM patients with CD4 counts > 200 as compared to HIV-negative MSM. However, they did not show any comparison between HIV-positive MSM (ART, CD4 >200) and HIV-negative non–MSM [43]. We did not observe any such trends for Anelloviruses between PWH on ART with CD4 counts >200 and controls, possibly because of the exclusion of homosexuals in our study. The same plasma study on MSM has also reported positive and negative correlations of Anellovirus with HIV viral load and CD4 count, respectively, in HIV-positive ART-naïve MSM [43]. We did not observe any such correlation because the PWH included in our study were on suppressive ART for more than 1.5 years.

The limitations of this study are small sample size, low sample volume, absence of treatment naïve or T-cell depleted HIV patients and less number of viral reads. Although Virus-like particle (VLP) enrichment ensures host-free extraction of virus DNA and random amplification before library preparation increases the amount of starting material required for library preparation and sequencing, they also cause unequal amplification of different DNA forms, with some viruses amplifying preferentially [85]. To avoid any such bias, our protocol did not include any VLP enrichment and pre-amplification step, which could have also resulted in a relatively low number of viral reads in our study. Furthermore, in this study, the cytokines and chemokines were quantified in plasma samples. However, measurement of these cytokines within T cells using intracellular staining would have enhanced the understanding about the relationship between T cell dysfunction and inflammation studied here. Due to the limited volume of blood collected (5-6 mL) from each subject, this aspect could not be included. The PBMCs were instead utilized for the identification of other markers in two different panels- one focused on senescence and the other on exhaustion. The PBMCs were fully utilized in these panels, leaving no cells available for additional experiments. While, this study faced challenges in obtaining sufficient samples for this additional analysis. Therefore, future studies can be done where intracellular cytokine expression within T cells can be examined.

## CONCLUSION

In conclusion, our findings have shown distinct plasma virome profiles in PWH on ART. The difference is thought to be driven by a differentially abundant viral species *Human gammaherpesvirus 4*. In addition, it correlated directly with the exhaustion phenotype of T cells and pro-inflammatory cytokine TNF-α. Hence, altered plasma virome during HIV infection can potentially lead to dysfunctional T cells and cause TLR-mediated inflammatory responses. Our pilot study is unique in a way that it revealed the virome composition in ART-suppressed HIV-infected Indian subjects for the first time. Similar kinds of studies that were conducted in the past on the treatment naïve HIV patients included individuals from other ethnic backgrounds. Future investigations with a larger sample size and advanced virus database will help us develop a broader perspective regarding the role of commensal viruses in modulating immune responses in PWH on ART.

## Figures and Tables

**Table 1:** Details of number, CD4 count, viral load and treatment regimen of study participants.

**Parameters**	**People with HIV (PWH) on ART**	**Uninfected Controls**	***p*-value**
Total number of samples	9	8	NA
Number of samples used for sequencing	6	6	NA
Number of samples used for multiplex cytokine assay	7	7	NA
Median Age (years (IQR)) for all samples	32 (28-45.5)	27.5 (22.5-37.25)	0.1377
Median Age (years (IQR)) for samples used in sequencing	36 (27-47.25)	27.5 (24.50-32.50)	0.1797
Median CD4^+^ T cell count (cells/mm^3^ (IQR))	386 (340.5-455.5)	NA	NA
Median HIV-1 viral load (RNA copies/mL (IQR))	Total load undetected	NA	NA
Race	Asian (from India)	Asian (from India)	NA
Gender of all samples	Male (66.67%)	Male (62.5%)	NA
-	Female (33.33%)	Female (37.5%)	-
Gender of samples used for sequencing	Male (66.67%)	Male (66.67%)	NA
-	Female (33.33%)	Female (33.33%)	-
Treatment regimen	TLE (7/9), ZLN (2/9)	NA	NA

**Abbreviations:** TLE = Tenofovir Lamivudine Efavirenz

ZLN = Zidovudine Lamivudine Nevirapine

NA = Not applicable

## Data Availability

Sequence data has been submitted to Mendeley data (DOI: 10.17632/tk4f76sxn4.2).

## LIST OF ABBREVIATIONS

COPD: Chronic Obstructive Pulmonary Disease
DA: Differentially Abundant
DM: Diabetes Mellitus
EBV: Epstein-Barr Virus
FCSB: Flow Cytometry Staining Buffer
FMO: Fluorescence Minus One
IBSC: Institutional Biosafety Committee
ICIs: Immune Checkpoint Inhibitors
IDUs: Intravenous Drug Users
IERB: Institutional Ethics Review Board
IRF: Immune Recovery Folliculitis
JNU: Jawaharlal Nehru University
KGMU: King George’s Medical University
NACO: National AIDS Control Organisation
PCoA: Principal Coordinate Analysis
PFA: Paraformaldehyde
PWH: People with HIV
TSS: Total Sum Scaling
TTV: *Torque teno virus*
VLP: Virus-like Particle

## STANDARD OF REPORTING

STROBE guidelines were followed.
