# Supplementary material for: Plasma Virome of HIV-infected Subjects on Suppressive Antiretroviral Therapy Reveals Association of Differentially Abundant Viruses with Distinct T-cell Phenotypes and Inflammation
Source: Curr Genomics. 2024 Jan 22;25(2):105–19. doi: 10.2174/0113892029279786240111052824 (PMC11092910; doi:10.2174/0113892029279786240111052824)
Supplement: Supplementary file 1 [file CG-25-105_SD1.pdf]

Supplementary Material

Plasma Virome of HIV-infected Subjects on Suppressive Antiretroviral Therapy Reveals Association of Differentially Abundant Viruses with Distinct T-cell Phenotypes and Inflammation

Tannu Bhagchandani[1], Mohammad M.U. Haque[2], Shilpa Sharma[3], Md Zubbair Malik[4], Ashwini Kumar Ray[5], Urvinder S. Kaur[1], Ankita Rai[1], Anjali Verma[1], Kamal Kumar Sawlani[6], Rupesh Chaturvedi[4,9], Himanshu Dandu[6], Abhishek Kumar[7,8] and Ravi Tandon[1,*]

[1]Laboratory of AIDS Research and Immunology, School of Biotechnology, Jawaharlal Nehru University, New Delhi, India; [2]School of Computational and Integrative Sciences, Jawaharlal Nehru University, New Delhi, India; [3]School of Biotechnology, Jawaharlal Nehru University, New Delhi, India; [4]Host-Pathogen Interaction Laboratory, School of Biotechnology, Jawaharlal Nehru University, New Delhi, India; [5]Laboratory of Metabolic Disorder and Environmental Biotechnology, Department of Environmental Studies, Faculty of Science, University of Delhi, New Delhi, India; [6]Department of Medicine, King George's Medical University, Lucknow, India; [7]Institute of Bioinformatics, International Technology Park, Bangalore; India; [8]Manipal Academy of Higher Education (MAHE), Manipal, India; [9]Special Centre for System Medicine, Jawaharlal Nehru University, New Delhi, India

Table S1. Table of subject characteristics and demographics.

S. No.	Subject Group	Age (years)	Gender	Race	Length of time on ART	ART regimen	CD4 count (cells/uL)	Plasma viral load of HIV (RNA copies/mL)	Experiments performed
01.	HIV- infected	24	Male	Asian (India)	>4 years	TLE	361	not detected	sequencing, immunophenotyping, luminex
02.	HIV- infected	28	Male	Asian (India)	>3 years	TLE	386	not detected	immunophenotyping
03.	HIV- infected	28	Male	Asian (India)	>2 years	TLE	336	not detected	sequencing, immunophenotyping, luminex
04.	HIV- infected	32	Male	Asian (India)	>2 years	TLE	458	not detected	sequencing, immunophenotyping, luminex
05.	HIV- infected	51	Male	Asian (India)	>1.5 years	TLE	606	not detected	sequencing, immunophenotyping, luminex
06.	HIV- infected	46	Female	Asian (India)	>7 years	ZLN	493	not detected	sequencing, immunophenotyping, luminex
07.	HIV- infected	40	Female	Asian (India)	>6 years	TLE	383	not detected	sequencing, immunophenotyping, luminex
08.	HIV- infected	45	Female	Asian (India)	>1.5 years	ZLN	402	not detected	Immunophenotyping
09.	HIV- infected	28	Male	Asian (India)	>3 years	TLE	345	not detected	immunophenotyping, luminex
10.	Uninfected control	37	Male	Asian (India)	NA	NA	NA	NA	sequencing, immunophenotyping, luminex
11.	Uninfected control	26	Male	Asian (India)	NA	NA	NA	NA	sequencing, immunophenotyping, luminex
12.	Uninfected control	22	Male	Asian (India)	NA	NA	NA	NA	immunophenotyping
13.	Uninfected control	31	Female	Asian (India)	NA	NA	NA	NA	sequencing, immunophenotyping, luminex
14.	Uninfected control	29	Female	Asian (India)	NA	NA	NA	NA	sequencing, immunophenotyping, luminex
15.	Uninfected control	34	Female	Asian (India)	NA	NA	NA	NA	immunophenotyping, luminex
16.	Uninfected control	25	Male	Asian (India)	NA	NA	NA	NA	sequencing, immunophenotyping, luminex
17.	Uninfected control	23	Male	Asian (India)	NA	NA	NA	NA	sequencing, immunophenotyping, luminex

TLE = tenofovir + lamivudine + efavirenz

ZLN = zidovudine + lamivudine + Nevirapine

NA = Not applicable

Fig. S1. Layout representing gating strategy for identifying the senescence phenotype of T cells.

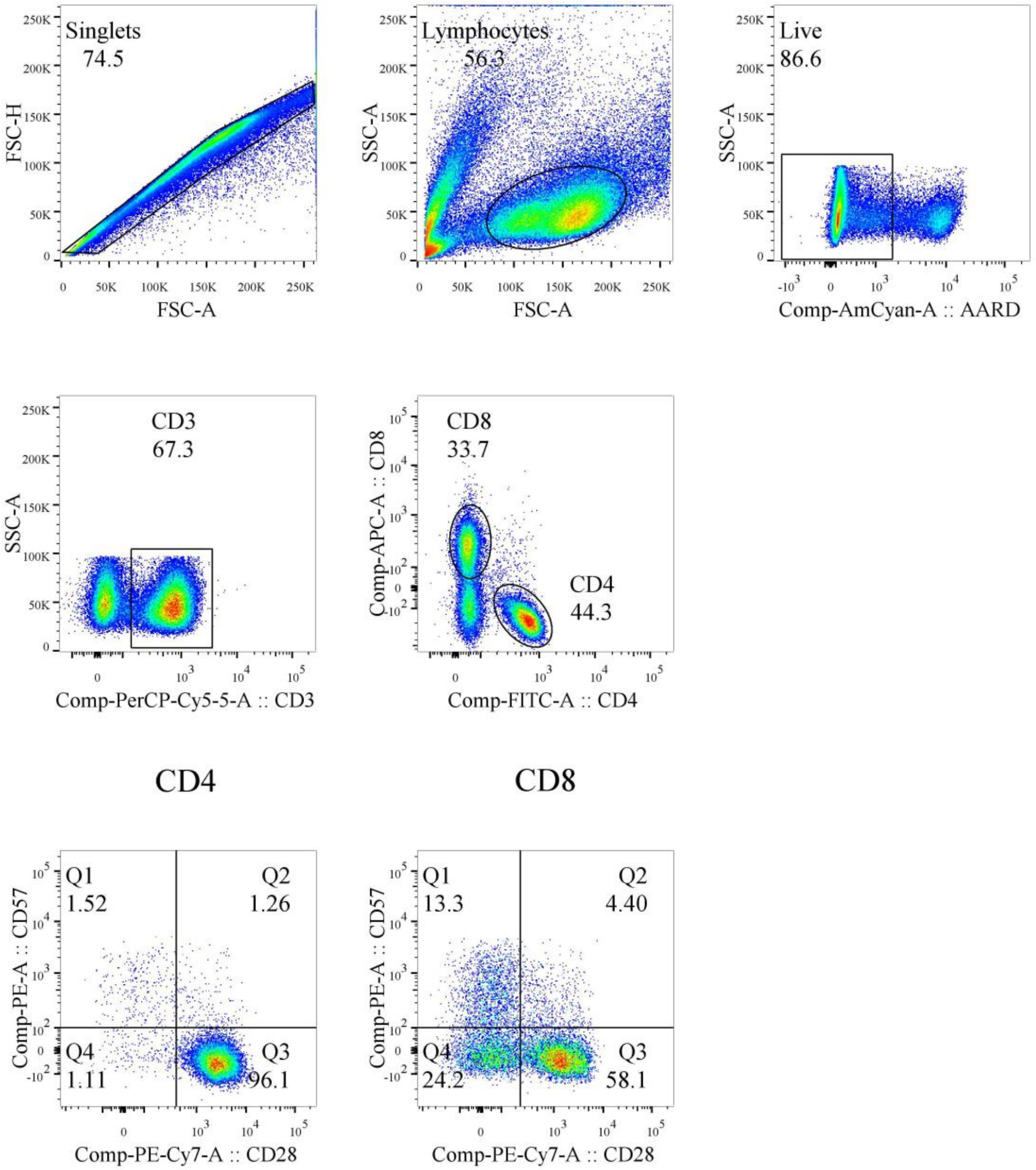

Fig. S2. Layout representing gating strategy for identifying the exhaustion phenotype of T cells.

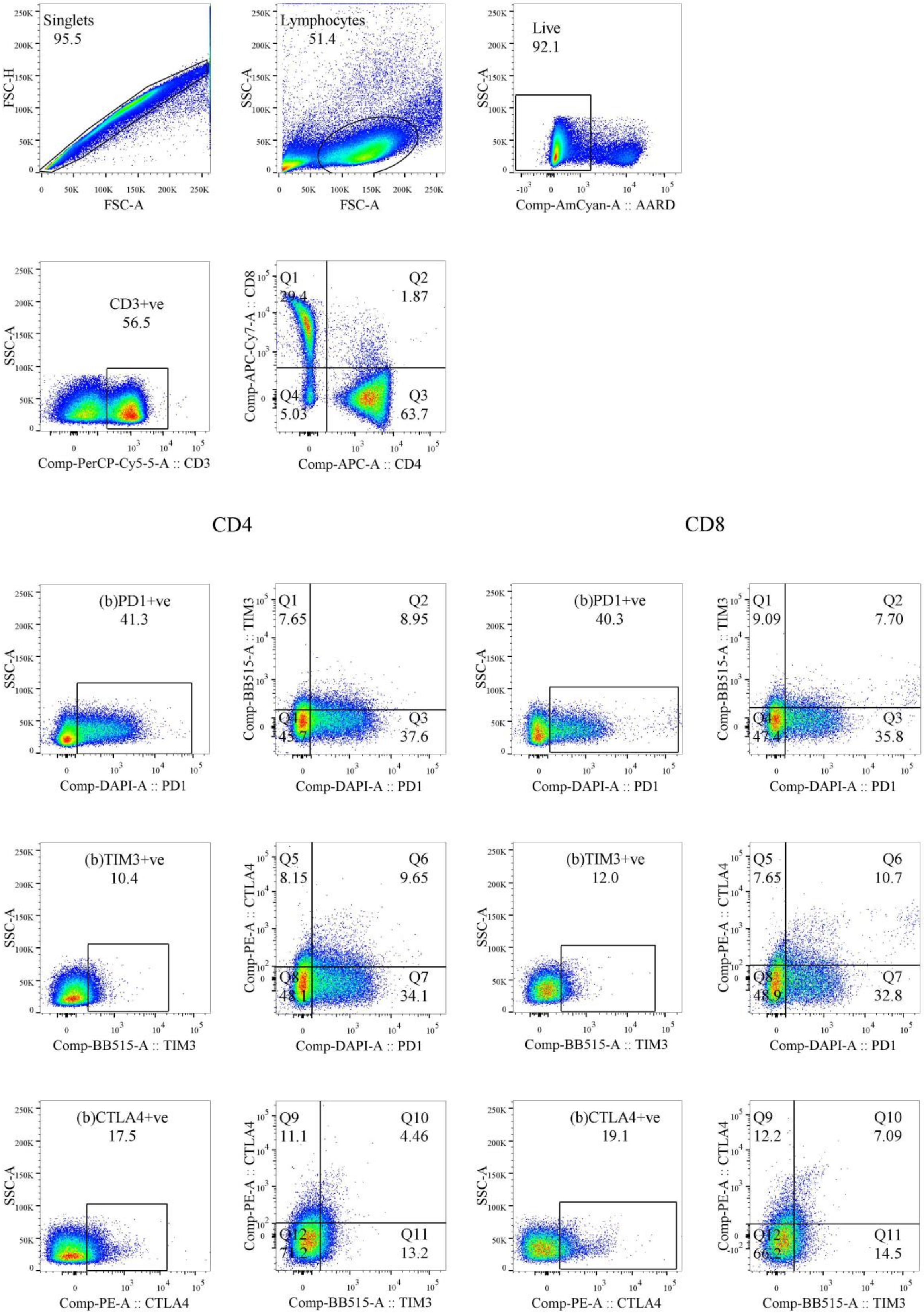

Fig. S3. Heatmaps showing the relative abundance and overall composition of (A) prokaryotic and (B) eukaryotic viral species present across all individuals.

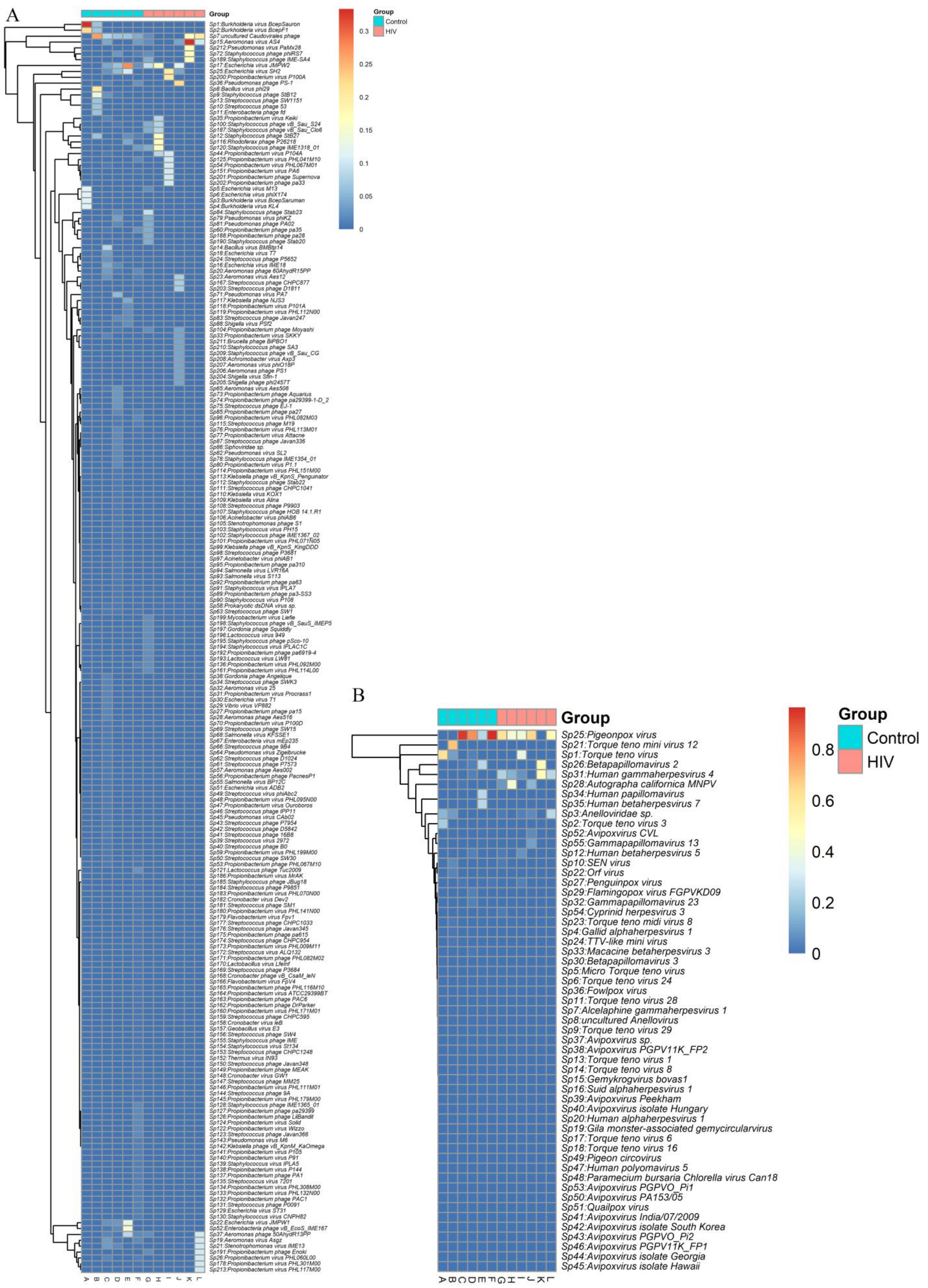

Fig. S4. Stacked bar plots showing the relative abundance of (A) prokaryotic and (B) eukaryotic viral families present in PWH on ART and uninfected controls. Heatmaps showing the relative abundance and overall composition of (C) prokaryotic and (D) eukaryotic viral families present across all individuals. Ordination plots representing beta diversity of (E) prokaryotic (R^2=0.15444, p=0.037*) and (F) eukaryotic (R^2=0.15444, p=0.037*) viral families.

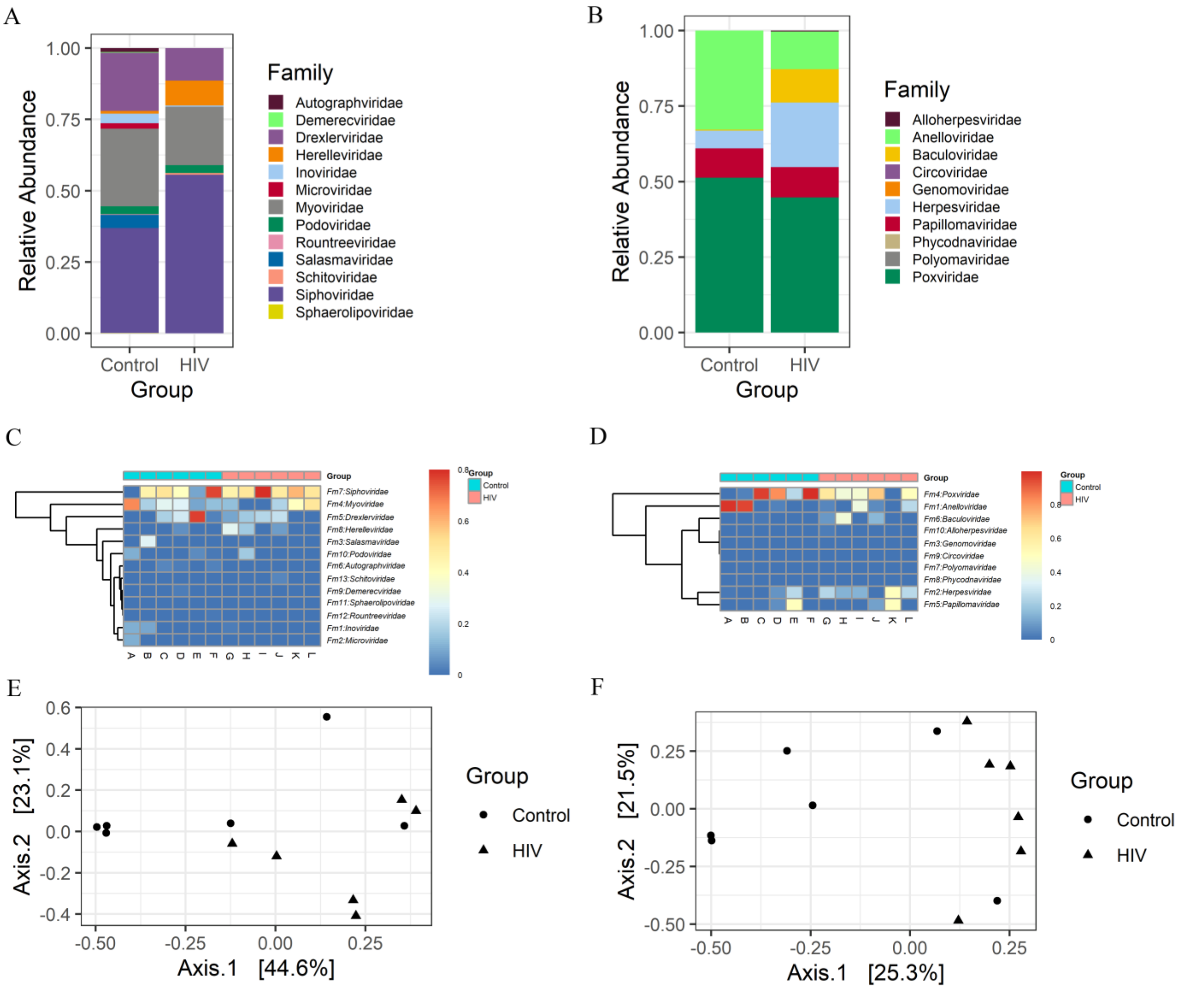

Table S2. List of differentially abundant viral species analyzed using ANCOM-BC. Here W denotes test statistic.

Markers	Feature	Type	Group	W	P value
Marker1	*Staphylococcus phage vB_Sau_Clo6*	Prokaryotic virus	HIV	3.322129	0.000893
Marker2	*Staphylococcus phage IME-SA4*	Prokaryotic virus	HIV	3.177651	0.001485
Marker3	*Propionibacterium phage Enoki*	Prokaryotic virus	HIV	3.482006	0.000498
Marker1	*Human gammaherpesvirus 4*	Eukaryotic virus	HIV	3.261829	0.007749
Marker2	*Cyprinid herpesvirus 3*	Eukaryotic virus	HIV	3.960543	0.012689
Marker3	*Avipoxvirus CVL*	Eukaryotic virus	HIV	2.997815	0.001047

Fig. S5. Box plots representing difference in the (A) percentage of CD57, (B) CTLA4, (C) PD1 and, (D) TIM3 on T cell population (CD4 and CD8 T cell) between uninfected controls and PWH on ART. The X-axis shows the different groups - control and HIV. The Y-axis shows the percentage of markers on the T cells. The median values of the percentage of markers CD57, CTLA4, PD1 and TIM3 in controls and PWH are The median values of the percentage of markers CD57, CTLA4, PD1 and TIM3 in controls and PWH are (8.477%; 7.8, 13.76 *versus* 18.95%; 10.47, 36.36), (19.06%; 5.528, 21.48 *versus* 16.89%; 14.75, 25.71), (26.80%; 23.05, 31.37 *versus* 28.08%; 23.61, 36.59) and (25.61%; 19.34, 39.14 *versus* 29.11%; 18.38, 38.84), respectively. A significant increase in the percentage of CD57 marker on T cell population was observed in PWH on ART as compared to controls. Mann-Whitney-Wilcoxon test was used to evaluate statistical significance, p<0.05 was considered to be significant.

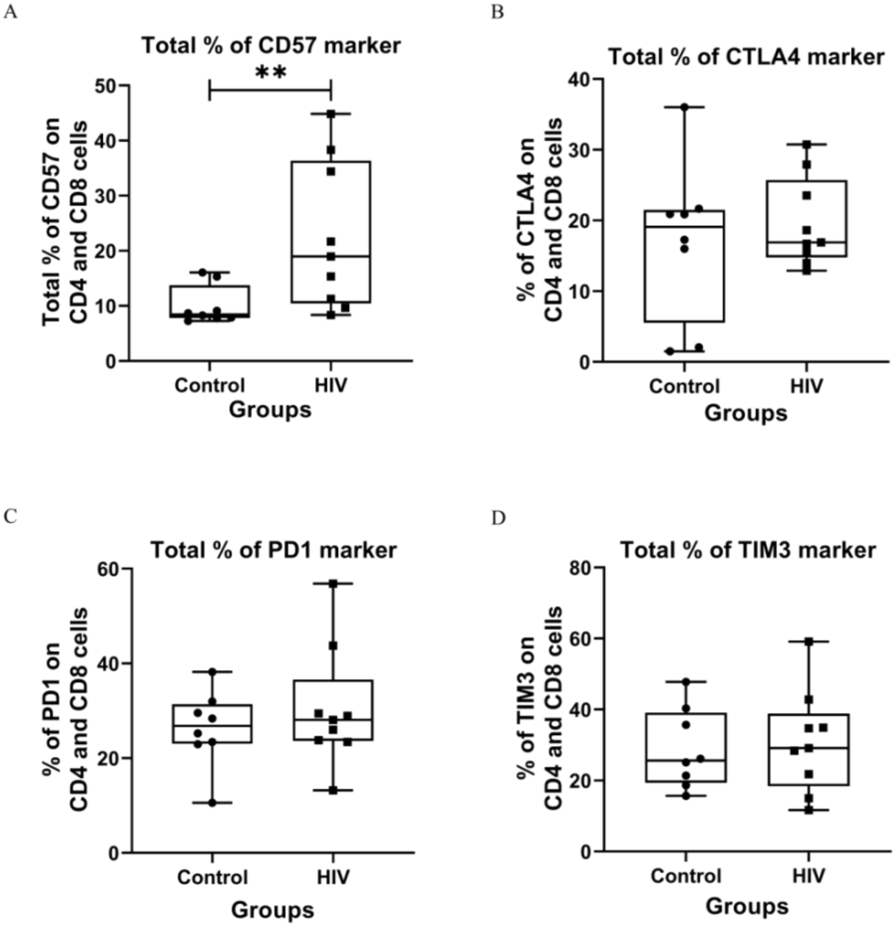

Table S3. List of significant correlations of prokaryotic viral species with T cell subsets and cytokines in HIV patients on ART. Here R is the Spearman Rank correlation coefficient.

Viral Species	Marker	R	p value
Escherichia virus JMPW2	$CTLA4^-$ $PD1^+$ $CD4^+$ T cells	-0.81969	0.045837
Escherichia virus JMPW2	$CTLA4^+$ $PD1^-$ $CD4^+$ T cells	0.880406	0.020599
Escherichia virus JMPW2	$CTLA4^-$ $TIM3^+$ $CD4^+$T cells	-0.81969	0.045837
Escherichia virus JMPW2	$CTLA4^+$ $PD1^-$ $TIM3^-$ $CD4^+$ T cells	0.880406	0.020599
Escherichia virus JMPW2	$CTLA4^-$ $PD1^+$ $TIM3^-$ $CD4^+$ T cells	-0.81969	0.045837
Escherichia virus SH2	$CTLA4^+$ $CD4^+$ T cells	-0.84515	0.034109
Escherichia virus SH2	$CTLA4^+$ $CD8^+$ T cells	-0.84515	0.034109
Aeromonas phage 50AhydR13PP	$PD1^-$ $TIM3^+$ $CD4^+$ T cells	0.845154	0.034109
Aeromonas phage 50AhydR13PP	$CTLA4^-$ $PD1^-$ $TIM3^+$ $CD4^+$ T cells	0.845154	0.034109
Aeromonas phage 50AhydR13PP	$CTLA4^-$ $TIM3^+$ $CD8^+$ T cells	0.845154	0.034109
Propionibacterium virus P104A	$CTLA4^+$ $PD1^+$ $TIM3^+$ $CD4^+$ T cells	-0.84515	0.034109
Staphylococcus phage phiRS7	Hu MIP-1β	-0.84515	0.034109
Staphylococcus phage phiRS7	Hu TNF-α	0.845154	0.034109
Staphylococcus phage phiRS7	$CD28^-$ $CD4^+$ T cells	0.845154	0.034109
Staphylococcus phage phiRS7	$CD57^+$ $CD4^+$ T cells	0.845154	0.034109
Staphylococcus phage phiRS7	$CD28^-CD57^+$ $CD4^+$ T cells	0.845154	0.034109
Staphylococcus phage phiRS7	$PD1^+$ $CD4^+$ T cells	0.857493	0.029015
Staphylococcus phage phiRS7	$CTLA4^+PD1^+TIM3^-$ $CD4^+$ T cells	0.845154	0.034109
Staphylococcus phage vB_Sau_S24	$CTLA4^+PD1^-$ $CD4^+$ T cells	0.845154	0.034109
Staphylococcus phage vB_Sau_S24	$CTLA4^+TIM3^-$ $CD4^+$ T cells	0.845154	0.034109
Staphylococcus phage vB_Sau_S24	$CTLA4^+PD1^-TIM3^-$ $CD4^+$ T cells	0.845154	0.034109
Staphylococcus phage vB_Sau_S24	$CTLA4^+$ $CD8^+$ T cells	0.845154	0.034109
Staphylococcus phage vB_Sau_S24	$CTLA4^+$ $PD1^+$ $TIM3^+$ $CD8^+$ T cells	0.845154	0.034109
Staphylococcus phage vB_Sau_S24	$CTLA4^+$ $PD1^-$ $TIM3^-$ $CD8^+$ T cells	0.845154	0.034109
Staphylococcus phage IME1318_01	$CTLA4^+$ $PD1^-$ $CD4^+$ T cells	0.845154	0.034109
Staphylococcus phage IME1318_01	$CTLA4^+$ $TIM3^-$ $CD4^+$ T cells	0.845154	0.034109
Staphylococcus phage IME1318_01	$CTLA4^+$ $PD1^-$ $TIM3^-$ $CD4^+$ T cells	0.845154	0.034109
Staphylococcus phage IME1318_01	$CTLA4^+$ $CD8^+$ T cells	0.845154	0.034109
Staphylococcus phage IME1318_01	$CTLA4^+$ $PD1^+$ $TIM3^+$ $CD8^+$ T cells	0.845154	0.034109
Staphylococcus phage IME1318_01	$CTLA4^+$ $PD1^-$ $TIM3^-$ $CD8^+$ T cells	0.845154	0.034109
Staphylococcus phage vB_Sau_Clo6	$CTLA4^+$ $PD1^-$ $CD4^+$ T cells	0.845154	0.034109
Staphylococcus phage vB_Sau_Clo6	$CTLA4^+$ $TIM3^-$ $CD4^+$ T cells	0.845154	0.034109
Staphylococcus phage vB_Sau_Clo6	$CTLA4^+$ $PD1^-$ $TIM3^-$ $CD4^+$ T cells	0.845154	0.034109
Staphylococcus phage vB_Sau_Clo6	$CTLA4^+$ $CD8^+$ T cells	0.845154	0.034109
Staphylococcus phage vB_Sau_Clo6	$CTLA4^+$ $PD1^+$ $TIM3^+$ $CD8^+$ T cells	0.845154	0.034109
Staphylococcus phage vB_Sau_Clo6	$CTLA4^+$ $PD1^-$ $TIM3^-$ $CD8^+$ T cells	0.845154	0.034109
Staphylococcus phage IME-SA4	Hu MIP-1β	-0.84515	0.034109
Staphylococcus phage IME-SA4	Hu TNF-α	0.845154	0.034109
Staphylococcus phage IME-SA4	$CD28^-$ $CD4^+$ T cells	0.845154	0.034109
Staphylococcus phage IME-SA4	$CD57^+$ $CD4^+$ T cells	0.845154	0.034109
Staphylococcus phage IME-SA4	$CD28^-$ $CD57^+$ $CD4^+$ T cells	0.845154	0.034109

Staphylococcus phage IME-SA4	$PD1^+ CD4^+$ T cells	0.857493	0.029015
Staphylococcus phage IME-SA4	$CTLA4^+ PD1^+ TIM3^- CD4^+$ T cells	0.845154	0.034109
Propionibacterium phage Enoki	$PD1^- TIM3^+ CD4^+$ T cells	0.845154	0.034109
Propionibacterium phage Enoki	$CTLA4^- PD1^- TIM3^+ CD4^+$ T cells	0.845154	0.034109
Propionibacterium phage Enoki	$CTLA4^- TIM3^+ CD8^+$ T cells	0.845154	0.034109

Table S4. List of significant correlations of eukaryotic viral species with T cell subsets and cytokines in HIV patients on ART. Here R is the Spearman Rank correlation coefficient.

**Viral Species**	**Marker**	**R**	**p value**
Anelloviridae sp.	Hu IL-8	0.927634	0.007666
Anelloviridae sp.	$CTLA4^- PD1^- TIM3^+ CD4^+$ T cells	0.811679	0.049858
Anelloviridae sp.	$CTLA4^+ TIM3^- CD8^+$ T cells	-0.81168	0.049858
Autographa californica MNPV	$CTLA4^- PD1^+ CD4^+$ T cells	-0.81969	0.045837
Autographa californica MNPV	$CTLA4^+ PD1^- CD4^+$ T cells	0.880406	0.020599
Autographa californica MNPV	$CTLA4^- TIM3^+ CD4^+$ T cells	-0.81969	0.045837
Autographa californica MNPV	$CTLA4^+ PD1^- TIM3^- CD4^+$ T cells	0.880406	0.020599
Autographa californica MNPV	$CTLA4^- PD1^+ TIM3^- CD4^+$ T cells	-0.81969	0.045837
Human gammaherpesvirus 4	Hu MIP-1β	-0.81969	0.045837
Human gammaherpesvirus 4	Hu TNF-α	0.819689	0.045837
Human gammaherpesvirus 4	$PD1^+ CD4^+$ T cells	0.831655	0.040124
Human gammaherpesvirus 4	$CTLA4^+ PD1^+ CD4^+$ T cells	0.880406	0.020599
Human gammaherpesvirus 4	$CTLA4^+ PD1^+ TIM3^- CD4^+$ T cells	0.819689	0.045837

Table S5. List of significant correlations between T cell subsets and cytokines in HIV patients on ART. Here R is the Spearman Rank correlation coefficient.

**Cytokine**	**Immune cell**	**R**	**p value**
Hu IL-8	$CD4^+CTLA4^-PD1^-TIM3^+$	0.942857	0.004805
Hu MIP-1β	$CD4^+CD28^-$	-0.94286	0.004805
Hu MIP-1β	$CD4^+CD28^-CD57^+$	-0.82857	0.041563
Hu MIP-1β	$CD4^+PD1^+$	-0.98561	0.000309
Hu MIP-1β	$CD4^+CTLA4^-PD1^+$	-0.88571	0.018845
Hu MIP-1β	$CD4^+CTLA4^+PD1^+$	-0.88571	0.018845
Hu MIP-1β	$CD4^+PD1^+TIM3^-$	-0.94286	0.004805
Hu MIP-1β	$CD4^+CTLA4^-PD1^+TIM3^-$	-0.88571	0.018845
Hu MIP-1β	$CD8^+CD28^-CD57^+$	-0.88571	0.018845
Hu TNF-α	$CD4^+CD28^-$	0.828571	0.041563
Hu TNF-α	$CD4^+PD1^+$	0.985611	0.000309
Hu TNF-α	$CD4^+CTLA4^-PD1^+$	0.942857	0.004805
Hu TNF-α	$CD4^+CTLA4^+PD1^+$	0.828571	0.041563
Hu TNF-α	$CD4^+PD1^+TIM3^-$	0.885714	0.018845
Hu TNF-α	$CD4^+CTLA4^-PD1^+TIM3^-$	0.942857	0.004805

Table S6. List of significant correlations of prokaryotic viral species with T cell subsets and cytokines in uninfected controls. Here R is the Spearman Rank correlation coefficient.

**Viral species**	**Marker**	**R**	**P value**
Burkholderia virus BcepSauron	Hu MIP-1β	0.845154	0.034109
Burkholderia virus BcepSauron	$CD28^{-}$ $CD4^{+}$ T cells	0.845154	0.034109
Burkholderia virus BcepSauron	$CD28^{-}$ $CD57^{+}$ $CD4^{+}$ T cells	0.845154	0.034109
Burkholderia virus BcepSauron	$PD1^{+}$ $CD4^{+}$ T cells	0.845154	0.034109
Burkholderia virus BcepSauron	$CTLA4^{+}$ $PD1^{+}$ $CD4^{+}$ T cells	0.845154	0.034109
Burkholderia virus BcepSauron	$CTLA4^{+}$ $TIM3^{-}$ $CD4^{+}$ T cells	0.845154	0.034109
Burkholderia virus BcepSauron	$CTLA4^{+}$ $PD1^{+}$ $CD8^{+}$ T cells	0.845154	0.034109
Burkholderia virus BcepSauron	$CTLA4^{-}$ $TIM3^{+}$ $CD8^{+}$ T cells	-0.84515	0.034109
Burkholderia virus BcepSauron	$PD1^{-}$ $TIM3^{+}$ $CD8^{+}$ T cells	-0.85749	0.029015
Burkholderia virus BcepF1	Hu MIP-1β	0.845154	0.034109
Burkholderia virus BcepF1	$CD28^{-}$ $CD4^{+}$ T cells	0.845154	0.034109
Burkholderia virus BcepF1	$CD28^{-}$ $CD57^{+}$ $CD4^{+}$ T cells	0.845154	0.034109
Burkholderia virus BcepF1	$PD1^{+}$ $CD4^{+}$ T cells	0.845154	0.034109
Burkholderia virus BcepF1	$CTLA4^{+}$ $PD1^{+}$ $CD4^{+}$ T cells	0.845154	0.034109
Burkholderia virus BcepF1	$CTLA4^{+}$ $TIM3^{-}$ $CD4^{+}$ T cells	0.845154	0.034109
Burkholderia virus BcepF1	$CTLA4^{+}$ $PD1^{+}$ $CD8^{+}$ T cells	0.845154	0.034109
Burkholderia virus BcepF1	$CTLA4^{-}$ $TIM3^{+}$ $CD8^{+}$ T cells	-0.84515	0.034109
Burkholderia virus BcepF1	$PD1^{-}$ $TIM3^{+}$ $CD8^{+}$ T cells	-0.85749	0.029015
Stenotrophomonas virus IME13	$CTLA4^{-}$ $PD1^{+}$ $CD4^{+}$ T cells	0.941124	0.005098
Stenotrophomonas virus IME13	$CTLA4^{+}$ $PD1^{+}$ $CD4^{+}$ T cells	-0.81969	0.045837
Stenotrophomonas virus IME13	$CTLA4^{+}$ $TIM3^{+}$ $CD4^{+}$ T cells	-0.94112	0.005098
Stenotrophomonas virus IME13	$CTLA4^{+}$ $TIM3^{-}$ $CD4^{+}$ T cells	-0.81969	0.045837
Stenotrophomonas virus IME13	$CTLA4^{-}$ $PD1^{+}$ $TIM3^{-}$ $CD4^{+}$ T cells	0.819689	0.045837
Stenotrophomonas virus IME13	$CTLA4^{+}$ $PD1^{-}$ $CD8^{+}$ T cells	-0.81969	0.045837
Stenotrophomonas virus IME13	$CTLA4^{+}$ $TIM3^{+}$ $CD8^{+}$ T cells	-0.88041	0.020599
Enterobacteria phage vB_EcoS_IME167	$CD28^{-}$ $CD4^{+}$ T cells	-0.81969	0.045837
Enterobacteria phage vB_EcoS_IME167	$CD57^{+}$ $CD4^{+}$ T cells	-0.88041	0.020599
Enterobacteria phage vB_EcoS_IME167	$CD28^{-}$ $CD57^{+}$ $CD4^{+}$ T cells	-0.88041	0.020599
Enterobacteria phage vB_EcoS_IME167	$CTLA4^{+}$ $PD1^{+}$ $CD8^{+}$ T cells	-0.88041	0.020599
Enterobacteria phage vB_EcoS_IME167	$PD1^{-}$ $TIM3^{+}$ $CD8^{+}$ T cells	0.831655	0.040124
Enterobacteria phage vB_EcoS_IME167	$CTLA4^{+}$ $PD1^{+}$ $TIM3^{+}$ $CD8^{+}$ T cells	-0.81969	0.045837
Streptococcus phage SW1	$CTLA4^{-}$ $PD1^{+}$ $CD4^{+}$ T cells	0.880406	0.020599
Streptococcus phage SW1	$CTLA4^{+}$ $TIM3^{+}$ $CD4^{+}$ T cells	-0.88041	0.020599
Streptococcus phage SW1	$CTLA4^{-}$ $PD1^{+}$ $TIM3^{+}$ $CD4^{+}$ T cells	0.819689	0.045837
Streptococcus phage SW1	$CTLA4^{+}$ $CD8^{+}$ T cells	-0.81969	0.045837
Streptococcus phage SW1	$CTLA4^{-}$ $TIM3^{+}$ $CD8^{+}$ T cells	0.819689	0.045837
Streptococcus phage SW1	$CTLA4^{+}$ $TIM3^{+}$ $CD8^{+}$ T cells	-0.94112	0.005098
Streptococcus phage SW1	$CTLA4^{+}$ $TIM3^{-}$ $CD8^{+}$ T cells	-0.81969	0.045837
Aeromonas virus Aes508	$CTLA4^{-}$ $PD1^{+}$ $CD4^{+}$ T cells	0.880406	0.020599
Aeromonas virus Aes508	$CTLA4^{+}$ $TIM3^{+}$ $CD4^{+}$ T cells	-0.88041	0.020599
Aeromonas virus Aes508	$CTLA4^{-}$ $PD1^{+}$ $TIM3^{+}$ $CD4^{+}$ T cells	0.819689	0.045837

Aeromonas virus Aes508	CTLA4$^+$ CD8$^+$ T cells	-0.81969	0.045837
Aeromonas virus Aes508	CTLA4$^-$ TIM3$^+$ CD8$^+$ T cells	0.819689	0.045837
Aeromonas virus Aes508	CTLA4$^+$ TIM3$^+$ CD8$^+$ T cells	-0.94112	0.005098
Aeromonas virus Aes508	CTLA4$^+$ TIM3$^-$ CD8$^+$ T cells	-0.81969	0.045837
Streptococcus phage Javan247	CD28$^-$ CD4$^+$ T cells	-0.84515	0.034109
Streptococcus phage Javan247	PD1$^-$ TIM3$^+$ CD4$^+$ T cells	0.845154	0.034109
Streptococcus phage Javan247	PD1$^+$ CD8$^+$ T cells	-0.84515	0.034109
Streptococcus phage Javan247	PD1$^+$ TIM3$^-$ CD8$^+$ T cells	-0.84515	0.034109
Streptococcus phage Javan247	CTLA4$^+$ PD1$^+$ TIM3$^+$ CD8$^+$ T cells	-0.84515	0.034109
Shigella virus PSf2	CD28$^-$ CD4$^+$ T cells	-0.84515	0.034109
Shigella virus PSf2	PD1$^-$ TIM3$^+$ CD4$^+$ T cells	0.845154	0.034109
Shigella virus PSf2	PD1$^+$ CD8$^+$ T cells	-0.84515	0.034109
Shigella virus PSf2	PD1$^+$ TIM3$^-$ CD8$^+$ T cells	-0.84515	0.034109
Shigella virus PSf2	CTLA4$^+$ PD1$^+$ TIM3$^+$ CD8$^+$ T cells	-0.84515	0.034109
Streptococcus phage M19	Hu MCP$^-$1	-0.85749	0.029015

Table S7. List of significant correlations of eukaryotic viral species with T cell subsets and cytokines in uninfected controls. Here R is the Spearman Rank correlation coefficient.

**Viral Species**	**Marker**	**R**	**P value**
Torque teno virus	PD1$^+$ CD4$^+$ T cells	0.819689	0.045837
Torque teno virus	PD1$^+$ TIM3$^+$ CD4$^+$ T cells	0.880406	0.020599
Torque teno virus	CTLA4$^+$ PD1$^+$ TIM3$^+$ CD4$^+$ T cells	0.880406	0.020599
Anelloviridae sp.	Hu MIP-1β	0.845154	0.034109
Anelloviridae sp.	PD1$^+$ CD4$^+$ T cells	0.845154	0.034109
Anelloviridae sp.	CTLA4$^+$ PD1$^+$ CD4$^+$ T cells	0.845154	0.034109
Anelloviridae sp.	CTLA4$^+$ TIM3$^-$ CD4$^+$ T cells	0.845154	0.034109
Anelloviridae sp.	CTLA4$^+$ PD1$^+$ CD8$^+$ T cells	0.845154	0.034109
Anelloviridae sp.	CTLA4$^-$ TIM3$^+$ CD8$^+$ T cells	-0.84515	0.034109
SEN virus	CTLA4$^+$ TIM3$^+$ CD4$^+$ T cells	0.845154	0.034109
SEN virus	PD1$^+$ TIM3$^+$ CD4$^+$ T cells	0.845154	0.034109
SEN virus	CTLA4$^+$ PD1$^+$ TIM3$^+$ CD4$^+$ T cells	0.845154	0.034109
SEN virus	CTLA4$^+$ TIM3$^+$ CD8$^+$ T cells	0.845154	0.034109
SEN virus	PD1$^+$ TIM3$^+$ CD8$^+$ T cells	0.845154	0.034109
SEN virus	CTLA4$^+$ PD1$^+$ TIM3$^+$ CD8$^+$ T cells	0.845154	0.034109
Betapapillomavirus 2	CD57$^+$ CD4$^+$ T cells	-0.84067	0.036058
Betapapillomavirus 2	PD1$^+$ CD4$^+$ T cells	-0.81168	0.049858
Betapapillomavirus 2	CTLA4$^+$ PD1$^+$ TIM3$^+$ CD4$^+$ T cells	-0.81168	0.049858
Betapapillomavirus 2	CTLA4$^+$ PD1$^+$ CD8$^+$ T cells	-0.92763	0.007666
Betapapillomavirus 2	CTLA4$^-$ TIM3$^+$ CD8$^+$ T cells	0.811679	0.049858
Betapapillomavirus 2	PD1$^+$ TIM3$^+$ CD8$^+$ T cells	-0.81168	0.049858
Betapapillomavirus 2	CTLA4$^+$ PD1$^+$ TIM3$^+$ CD8$^+$ T cells	-0.92763	0.007666

Table S8. List of significant correlations between T cell subsets and cytokines in uninfected controls. Here R is the Spearman Rank correlation coefficient.

Cytokine	Immune cell	R	P value
Hu IL-8	$PD1^- TIM3^+ CD4^+$ T cells	0.811679	0.049858
Hu IL-8	$CTLA4^+ PD1^- TIM3^+ CD4^+$ T cells	0.869657	0.024377
Hu IL-8	$CTLA4^- PD1^+ CD8^+$ T cells	-0.98561	0.000309
Hu IL-8	$PD1^+ TIM3^- CD8^+$ T cells	-0.81168	0.049858
Hu IL-8	$CTLA4^- PD1^+ TIM3^- CD8^+$ T cells	-0.92763	0.007666
Hu MIP-1β	$CD28^- CD4^+$ T cells	0.828571	0.041563
Hu MIP-1β	$CD28^- CD8^+$ T cells	0.885714	0.018845
Hu MIP-1β	$CTLA4^+ PD1^+ CD8^+$ T cells	0.828571	0.041563
Hu MIP-1β	$PD1^- TIM3^+ CD8^+$ T cells	-0.89865	0.014889

Fig. S6. Correlograms representing correlation patterns of prokaryotic and eukaryotic viral families with T cell immune phenotypes and inflammatory cytokines in HIV patients on ART (A and B, respectively) and in uninfected controls respectively (C and D, respectively). Spearman's Rank Correlation test was used to measure association between parameters on X and Y axis. Asterisks here are used to indicate correlations that are significant (p<0.05), P<0.05*, <0.01, <0.001***. Only statistically significant correlations were plotted, where blue circles denote direct correlations and red circles denote inverse correlations. The size and shading show the correlation's strength, with darker shades and larger circles denoting stronger correlations than light small ones.**

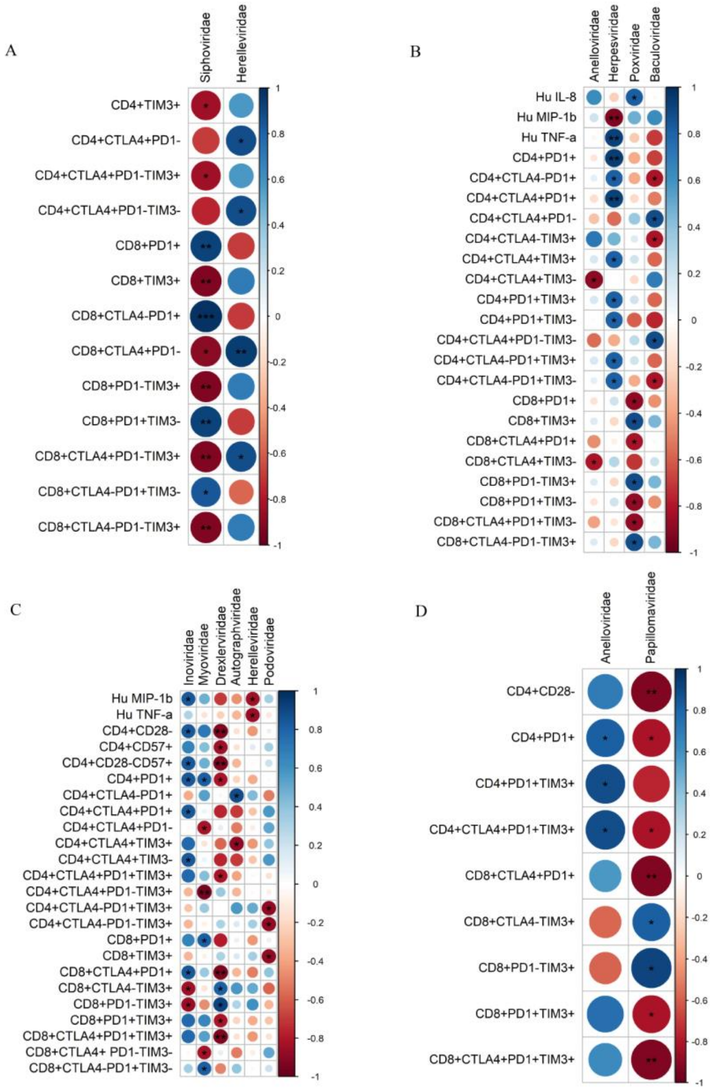

Table S9. List of significant correlations of prokaryotic viral families with T cell subsets and cytokines in HIV patients on ART. Here R is the Spearman Rank correlation coefficient.

Viral Family	Marker	R	p value
Siphoviridae	$TIM3^+$ $CD4^+$ T cells	-0.84067	0.036058
Siphoviridae	$CTLA4^+$ $PD1^-$ $TIM3^+$ $CD4^+$ T cells	-0.84067	0.036058
Siphoviridae	$PD1^+$ $CD8^+$ T cells	0.927634	0.007666
Siphoviridae	$TIM3^+$ $CD8^+$ T cells	-0.92763	0.007666
Siphoviridae	$CTLA4^-$ $PD1^+$ $CD8^+$ T cells	0.985611	0.000309
Siphoviridae	$CTLA4^+$ $PD1^-$ $CD8^+$ T cells	-0.89865	0.014889
Siphoviridae	$PD1^-$ $TIM3^+$ $CD8^+$ T cells	-0.92763	0.007666
Siphoviridae	$PD1^+$ $TIM3^-$ $CD8^+$ T cells	0.927634	0.007666
Siphoviridae	$CTLA4^+$ $PD1^-$$TIM3^+$ $CD8^+$ T cells	-0.92763	0.007666
Siphoviridae	$CTLA4^-$ $PD1^+$$TIM3^-$ $CD8^+$ T cells	0.840668	0.036058
Siphoviridae	$CTLA4^-$ $PD1^-$$TIM3^+$ $CD8^+$ T cells	-0.92763	0.007666
Herelleviridae	$CTLA4^+$ $PD1^-$ $CD4^+$ T cells	0.880406	0.020599
Herelleviridae	$CTLA4^+$ $PD1^-$ $TIM3^-$ $CD4^+$ T cells	0.880406	0.020599
Herelleviridae	$CTLA4^+$ $PD1^-$ $CD8^+$ T cells	0.941124	0.005098
Herelleviridae	$CTLA4^+$ $PD1^-$ $TIM3^+$ $CD8^+$ T cells	0.880406	0.020599

Table S10. List of significant correlations of eukaryotic viral families with T cell subsets and cytokines in HIV patients on ART. Here R is the Spearman Rank correlation coefficient.

Viral Family	Marker	R	p value
Anelloviridae	$CTLA4^+$ $TIM3^-$ $CD4^+$ T cells	-0.8986	0.01489
Anelloviridae	$CTLA4^+$ $TIM3^-$ $CD8^+$ T cells	-0.8117	0.04986
Herpesviridae	Hu MIP-1β	-0.9276	0.00767
Herpesviridae	Hu TNF-α	0.92763	0.00767
Herpesviridae	$PD1^+$ $CD4^+$ T cells	0.94118	0.00509
Herpesviridae	$CTLA4^-$ $PD1^+$ $CD4^+$ T cells	0.81168	0.04986
Herpesviridae	$CTLA4^+$ $PD1^+$ $CD4^+$ T cells	0.92763	0.00767
Herpesviridae	$CTLA4^+$ $TIM3^+$ $CD4^+$ T cells	0.81168	0.04986
Herpesviridae	$PD1^+$ $TIM3^+$ $CD4^+$ T cells	0.81168	0.04986
Herpesviridae	$PD1^+$ $TIM3^-$ $CD4^+$ T cells	0.81168	0.04986
Herpesviridae	$CTLA4^-$ $PD1^+$ $TIM3^+$ $CD4^+$ T cells	0.81168	0.04986
Herpesviridae	$CTLA4^-$ $PD1^+$ $TIM3^-$ $CD4^+$ T cells	0.81168	0.04986
Poxviridae	Hu IL-8	0.82857	0.04156
Poxviridae	$PD1^+$ $CD8^+$ T cells	-0.8857	0.01885
Poxviridae	$TIM3^+$ $CD8^+$ T cells	0.88571	0.01885
Poxviridae	$CTLA4^+$ $PD1^+$ $CD8^+$ T cells	-0.8286	0.04156
Poxviridae	$PD1^-$ $TIM3^+$ $CD8^+$ T cells	0.88571	0.01885
Poxviridae	$PD1^+$ $TIM3^-$ $CD8^+$ T cells	-0.8857	0.01885
Poxviridae	$CTLA4^+$ $PD1^+$ $TIM3^-$ $CD8^+$ T cells	-0.8857	0.01885
Poxviridae	$CTLA4^-$ $PD1^-$ $TIM3^+$ $CD8^+$ T cells	0.88571	0.01885
Baculoviridae	$CTLA4^-$ $PD1^+$ $CD4^+$ T cells	-0.8197	0.04584

Baculoviridae	CTLA4^{+} PD1^{-} CD4^{+} T cells	0.88041	0.0206
Baculoviridae	CTLA4^{-} TIM3^{+} CD4^{+} T cells	-0.8197	0.04584
Baculoviridae	CTLA4^{+} PD1^{-} TIM3^{-} CD4^{+} T cells	0.88041	0.0206
Baculoviridae	CTLA4^{-} PD1^{+} TIM3^{-} CD4^{+} T cells	-0.8197	0.04584

Table S11. List of significant correlations of prokaryotic viral families with T cell subsets and cytokines in uninfected controls. Here R is the Spearman Rank correlation coefficient.

Viral Family	Marker	R	p value
Inoviridae	Hu MIP-1β	0.84515	0.03411
Inoviridae	CD28^{-} CD4^{+} T cells	0.84515	0.03411
Inoviridae	CD28^{-} CD57^{+} CD4^{+} T cells	0.84515	0.03411
Inoviridae	PD1^{+} CD4^{+} T cells	0.84515	0.03411
Inoviridae	CTLA4^{+} PD1^{+} CD4^{+} T cells	0.84515	0.03411
Inoviridae	CTLA4^{+} TIM3^{-} CD4^{+} T cells	0.84515	0.03411
Inoviridae	CTLA4^{+} PD1^{+} CD8^{+} T cells	0.84515	0.03411
Inoviridae	CTLA4^{-} TIM3^{+} CD8^{+} T cells	-0.8452	0.03411
Inoviridae	PD1^{-} TIM3^{+} CD8^{+} T cells	-0.8575	0.02902
Myoviridae	PD1^{+} CD4^{+} T cells	0.82857	0.04156
Myoviridae	CTLA4^{+} PD1^{-} CD4^{+} T cells	-0.8286	0.04156
Myoviridae	CTLA4^{+} PD1^{-} TIM3^{+} CD4^{+} T cells	-0.9429	0.0048
Myoviridae	PD1^{+} CD8^{+} T cells	0.82857	0.04156
Myoviridae	CTLA4^{+} PD1^{-} TIM3^{-} CD8^{+} T cells	-0.8286	0.04156
Myoviridae	CTLA4^{-}PD1^{+}TIM3^{-} CD8^{+} T cells	0.82857	0.04156
Drexlerviridae	CD28^{-} CD4^{+} T cells	-0.9276	0.00767
Drexlerviridae	CD57^{+} CD4^{+} T cells	-0.8407	0.03606
Drexlerviridae	CD28^{-} CD57^{+} CD4^{+} T cells	-0.9276	0.00767
Drexlerviridae	PD1^{+} CD4^{+} T cells	-0.8117	0.04986
Drexlerviridae	CTLA4^{+} PD1^{+} TIM3^{+} CD4^{+} T cells	-0.8117	0.04986
Drexlerviridae	CTLA4^{+} PD1^{+} CD8^{+} T cells	-0.9276	0.00767
Drexlerviridae	CTLA4^{-} TIM3^{+} CD8^{+} T cells	0.81168	0.04986
Drexlerviridae	PD1^{-} TIM3^{+} CD8^{+} T cells	0.91176	0.01133
Drexlerviridae	PD1^{+} TIM3^{+} CD8^{+} T cells	-0.8117	0.04986
Drexlerviridae	CTLA4^{+} PD1^{+} TIM3^{+} CD8^{+} T cells	-0.9276	0.00767
Autographviridae	CTLA4^{-} PD1^{+} CD4^{+} T cells	0.88041	0.0206
Autographviridae	CTLA4^{+} TIM3^{+} CD4^{+} T cells	-0.8804	0.0206
Herelleviridae	Hu MIP-1β	-0.8452	0.03411
Herelleviridae	Hu TNF-α	-0.8575	0.02902
Podoviridae	CTLA4^{-} PD1^{+} TIM3^{+} CD4^{+} T cells	-0.8804	0.0206
Podoviridae	CTLA4^{-} PD1^{-} TIM3^{+} CD4^{+} T cells	-0.8804	0.0206
Podoviridae	TIM3^{+} CD8^{+} T cells	-0.8804	0.0206

Table S12. List of significant correlations of eukaryotic viral families with T cell subsets and cytokines in uninfected controls. Here R is the Spearman Rank correlation coefficient.

**Viral Family**	**Marker**	**R**	**p value**
Anelloviridae	$PD1^{+}$ $CD4^{+}$ T cells	0.81969	0.04584
Anelloviridae	$PD1^{+}$ $TIM3^{+}$ $CD4^{+}$ T cells	0.88041	0.0206
Anelloviridae	$CTLA4^{+}$ $PD1^{+}$ $TIM3^{+}$ $CD4^{+}$ T cells	0.88041	0.0206
Papillomaviridae	$CD28^{-}$ $CD4^{+}$ T cells	-0.9276	0.00767
Papillomaviridae	$PD1^{+}$ $CD4^{+}$ T cells	-0.8117	0.04986
Papillomaviridae	$CTLA4^{+}$ $PD1^{+}$ $TIM3^{+}$ $CD4^{+}$ T cells	-0.8117	0.04986
Papillomaviridae	$CTLA4^{+}$ $PD1^{+}$ $CD8^{+}$ T cells	-0.9276	0.00767
Papillomaviridae	$CTLA4^{-}$ $TIM3^{+}$ $CD8^{+}$ T cells	0.81168	0.04986
Papillomaviridae	$PD1^{-}$ $TIM3^{+}$ $CD8^{+}$ T cells	0.91176	0.01133
Papillomaviridae	$PD1^{+}$ $TIM3^{+}$ $CD8^{+}$ T cells	-0.8117	0.04986
Papillomaviridae	$CTLA4^{+}$ $PD1^{+}$ $TIM3^{+}$ $CD8^{+}$ T cells	-0.9276	0.00767
